# Understanding Solid-State Photochemical Energy Storage in Polymers with Azobenzene Side Groups

**DOI:** 10.1021/acsami.3c04631

**Published:** 2023-06-23

**Authors:** Callum Wallace, Kieran Griffiths, Benjamin L. Dale, Stuart Roberts, Jonathan Parsons, John M. Griffin, Verena Görtz

**Affiliations:** † Department of Chemistry, Lancaster University, Lancaster LA1 4YB, United Kingdom; ‡ Jaguar Land Rover Research, International Digital Laboratory, University of Warwick, Coventry CV4 7AL, United Kingdom; § Materials Science Institute, Lancaster University, Lancaster LA1 4YB, United Kingdom

**Keywords:** solar thermal fuel, azobenzene polymer, energy storage, solar energy, energy conversion, energy materials, photoresponsive polymer

## Abstract

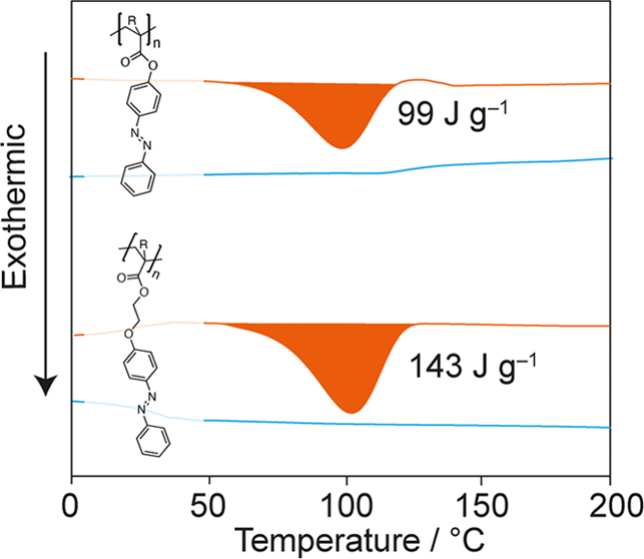

Solar thermal fuel (STF) materials store energy through light-induced changes in the structures of photoactive molecular groups, and the stored energy is released as heat when the system undergoes reconversion to the ground-state structure. Solid-state STF devices could be useful for a range of applications; however, the light-induced structural changes required for energy storage are often limited or prevented by dense molecular packing in condensed phases. Recently, polymers have been proposed as effective solid-state STF platforms, as they can offer the bulk properties of solid materials while retaining the molecular-level free volume and/or mobility to enable local structural changes in photoresponsive groups. Light-induced energy storage and macroscopic heat release have been demonstrated for polymers with photoisomerizable azobenzene side groups. However, the mechanism of energy storage and the link between the polymer structure, energy density and storage duration has not yet been explored in detail. In this work, we present a systematic study of methacrylate- and acrylate-based polymers with azobenzene side groups to establish the mechanism of energy storage and release and the factors affecting energy density and reconversion kinetics. For polymers with directly attached azobenzene side groups, the energy storage properties are in line with previous work on similar systems, and the photoisomerization and reconversion properties of the azobenzene side groups mirror those of molecular azobenzene. However, the inclusion of an alkyl linker between the azobenzene side group and the backbone significantly increases the photoswitching efficiency, giving almost quantitative conversion to the *Z* isomeric state. The presence of the alkyl linker also reduces the glass transition temperature and leads to faster spontaneous thermal reconversion to the *E* isomeric form, but in all cases, half-lives of more than 4 days are observed in the solid state, which provides scope for applications requiring daily energy storage–release cycles. The maximum gravimetric energy density observed is 143 J g^–1^, which represents an increase of up to 44% compared to polymers with directly attached azobenzene moieties.

## Introduction

The development of technologies for solar energy conversion and storage is crucial to support the global move toward renewable energy sources. One class of materials that attract increasing attention is solar thermal fuel (STF) energy storage materials, which use photoactive molecules to convert photon energy to thermal energy through reversible isomerization between ground and metastable isomeric states.^1,2^ A wide range of photoactive molecular systems have been studied for this purpose, including norbornadiene–quadricyclanes,^3,4^ fulvalene diruthenium,^5,6^ arylazopyrazoles,^7−9^ and azobenzenes.^10−14^ Of these, azobenzenes have received particular attention due to their high quantum yields, high-fatigue resistance, and appreciable energy separation between the ground-state *E* and metastable *Z* isomers.^15^ However, in their pure solid form, the photoisomerization of azobenzene derivatives and other photoswitches is often limited due to dense crystal packing. A number of strategies have been proposed to address this problem, including templating azobenzene functional groups on nanotubes^10,11,16^ and graphene,^17−19^ incorporation into frameworks,^13,14,20^ and the introduction of bulky functional groups to increase the free volume and prevent crystallization at ambient temperature.^12,21^ Another promising method is the attachment of azobenzene functional groups to polymers.^22−25^ This approach can offer access to photoswitchable solid-state materials with greater processability for coatings and devices while retaining comparatively high densities of photoactive groups and therefore high energy densities.

In a proof-of-concept study, Zhitomirsky et al. demonstrated that a simple methacrylate polymer with azobenzene side groups can store and release up to 104 J g^–1^.^26^ The polymer was shown to be solution processable into a uniform film coating for which a thermally triggered macroscopic energy release was demonstrated, resulting in a 10 °C temperature increase compared to an unirradiated film. However, to date, there has been limited fundamental insight into the mechanisms of photothermal energy storage in azobenzene-based polymers or the dominant factors in controlling the energy densities. In addition to spatial and steric considerations for allowing photoconversion between isomers, the degree of photoconversion to the metastable state is also a key factor. For azobenzene, the overlap of the π–π* absorption bands for the *E* and *Z* isomers means that the photostationary state (PSS) is limited to approximately 78% under 365 nm irradiation.^27^ One way to increase the intrinsic PSS is to incorporate functional groups, which alter the electronic structure to increase the absorption band separation for the *E* and *Z* isomers. This has been demonstrated for *ortho*-functionalized azobenzene derivatives for which the *n*–π* absorption bands are well separated for the *E* and *Z* isomers, leading to quantitative photoisomerization at visible wavelengths.^28−30^ However, while this approach can lead to more efficient photoisomerization, it can also reduce the energy difference between the ground and metastable states, thereby reducing the energy density of the STF material.

A further consideration is the stability of the metastable isomer. Azobenzene-based photoswitches will undergo spontaneous thermal reconversion from the metastable state to the ground-state isomer, typically via first-order kinetics controlled by the activation energy barrier between the two isomeric states. The rate of spontaneous thermal reconversion at ambient temperature (or equivalently the half-life of the metastable state) in the dark needs to be sufficiently long for the desired STF application. This will necessarily depend on the context in which the STF is to be used, but typically half-lives of at least several hours would be required for daily repeat cycles. Therefore, the structural design of STF materials needs to balance multiple factors regarding both spatial and electronic considerations to maximize the conversion to the metastable state as well as the energy density. This must be based on a detailed understanding of the photoisomerization properties and thermal energy storage and release mechanisms.

In this work, we present a systematic study of methacrylate- and acrylate-based polymers with azobenzene functional side groups as potential STF materials. We evaluate the absorption and photoisomerization properties of a set of six model polymers to understand the factors that control the photoisomerization efficiency, *Z* isomer stability, and the resulting energy density. We find that the inclusion of an alkoxy linker between the azobenzene side group and the backbone significantly increases the photoisomerization efficiency, giving almost quantitative conversion to the *Z* isomeric state. The presence of the alkoxy linker also reduces the glass transition temperature and leads to faster spontaneous thermal reconversion to the *E* isomeric form, but in all cases, half-lives of more than 4 days are observed in the solid state. The increased photoconversion in polymers with alkoxy linkers increases the energy density by up to 44% compared to polymers with directly attached azobenzene moieties. This is found to be primarily due to the increased absorption band separation of the ground and metastable isomeric states that results from this chemical modification, while structural effects including the reduced glass transition temperatures may also contribute to a lesser extent.

## Results and Discussion

### Structural Design of Photochromic Polymers

**Figure 1:**
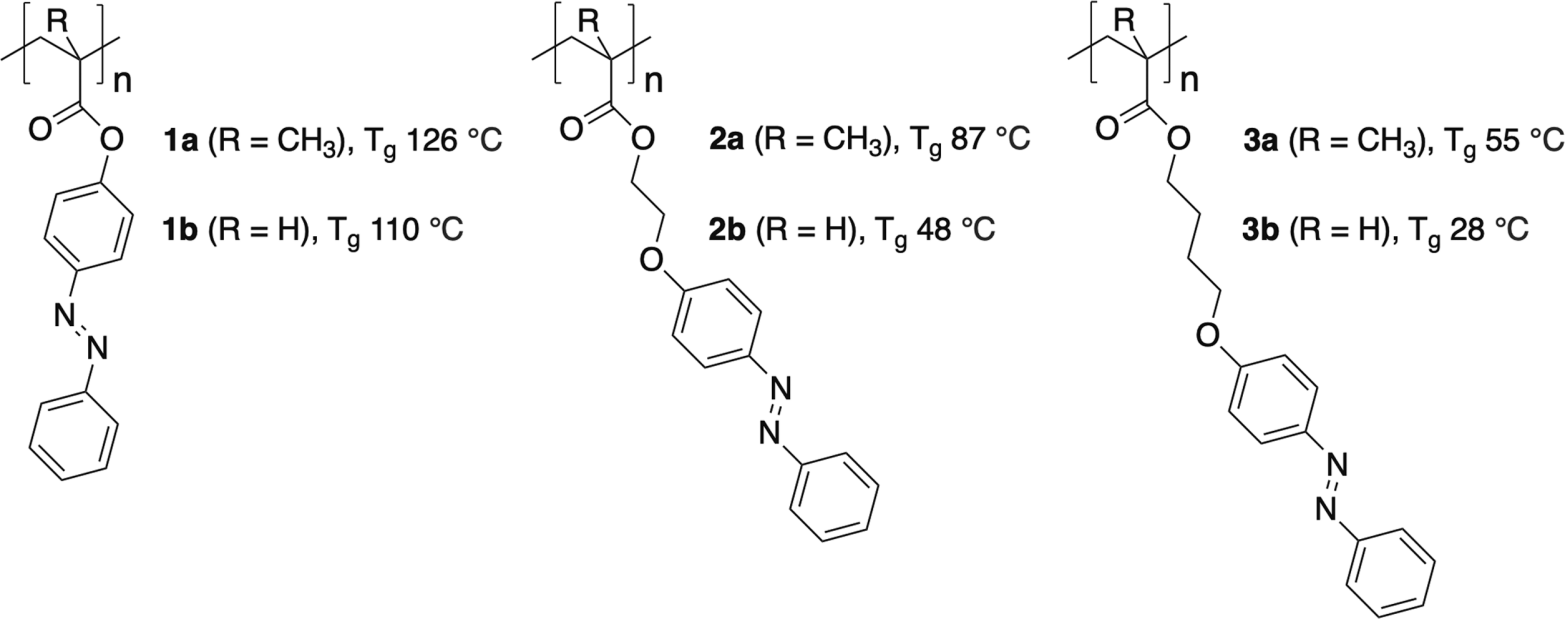
Structures and glass transition temperatures *T*_g_ of (meth)acrylate-based polymers investigated in this work.

The structures of the polymers studied in this work are shown in Figure 1. The basic polymer comprises an azobenzene side group directly bonded to either a methacrylate (**1a**) or acrylate (**1b**) backbone via the ester functionality.

To systematically investigate the influence of the side-chain structure on photoisomerization, we prepared analogues with flexible alkoxy linkers of varying lengths between the azobenzene group and the polymer backbone (**2a,b** and **3a,b**). All polymers were synthesized by free-radical polymerization in solution from monomers prepared in one step from 4-phenylazophenol, following slightly modified previously reported methodologies.^26,31,32^ The as-prepared polymers’ glass transition temperatures are shown in Figure 1. Detailed experimental procedures and analytical data can be found in the Supporting Information.

In side-chain functional polymers such as **1a**, the glass transition temperature, *T*_g_, which is a measure of polymer chain segment mobility, depends on the backbone structure, size, and nature of the attached side-chain functional groups and on the distance between these side-chain groups and the backbone, i.e., the length and structure of any linking groups present. Equally, the local mobility of the polymer can influence the ability of azobenzene functional groups to switch between the *E* and *Z* isomeric forms, which may also depend on the distance of the photochromic units to the backbone.^23^ In each polymethacrylate/acrylate pair **a**/**b**, methacrylate derivatives **a** have the higher *T*_g_. In both the series of polymethacrylates **1a**–**3a** and the series of polyacrylates **1b**–**3b**, *T*_g_ drops with increasing linker length (Figure S4). The combined effects of the removal of the methyl group and a successive increase in linker length result in a drop of *T*_g_ from 126 °C in **1a** over 48 °C in **2b** to 28 °C in **3b**.

### Photoisomerization in Solution

**Figure 2:**
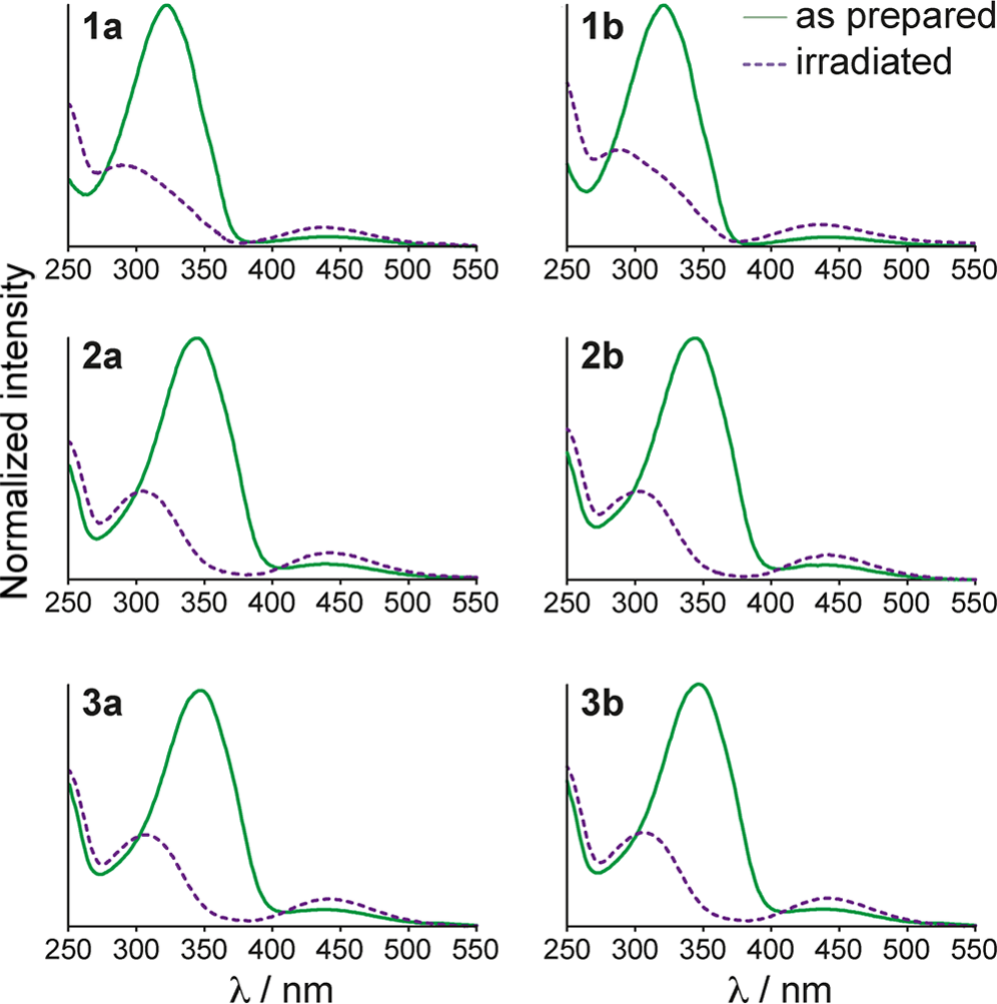
UV–vis absorption spectra for compounds **1a/b** (top), **2a/b** (middle), and **3a/b** (bottom) before (solid green line) and after (dashed purple line) irradiation with 365 nm light. All spectra were recorded in CH_2_Cl_2_. Spectra for each polymer are normalized to the maximum intensity of the as-prepared polymer (Table S1 for extinction coefficients ε at λ_max_).

To separate intrinsic molecular properties from any effects arising from solid-state morphologies, we investigated the UV–vis absorption and photoisomerization of the six polymers in solution. UV–vis spectra of dichloromethane solutions of the as-prepared polymers are shown in Figure 2. NMR analysis confirmed that all, or at least the vast majority of, azobenzene chromophores are in the *E* isomeric state under these conditions for all six polymers. Both **1a** and **1b** (Figure 2a,b) exhibit a strong π–π* absorption band at 325 nm and a weak *n*–π* band at 440 nm. The close similarity in absorption behavior between **1a** and **1b** is not surprising given that the only structural difference, the backbone methyl group, is minor and distant from the azobenzene moiety. The absorptions are also very similar to those obtained for both monomers and azobenzene itself, recorded under the same conditions, suggesting that attachment to the polymer backbone does not significantly affect the absorption properties of the azobenzene moiety.

However, for **2a**,**b** and **3a**,**b** (Figure 2), a significant shift of the absorption maximum for the π–π* transition from 325 nm to around 345 nm is observed. The same shifts are observed in the UV–vis spectra of the corresponding monomers (Figure S1), showing that this effect is a property of the side groups rather than the polymers. We attribute this bathochromic shift to the donation of electron density into the azobenzene moiety from the adjacent ether oxygen atom connected to the alkyl spacer.^27,33^ Conversely, in **1a** and **1b**, this oxygen is part of an ester group and therefore donates instead toward the more electron-deficient carbonyl carbon, and no significant shift in the π–π* transition relative to azobenzene is observed.

After irradiation with 365 nm light for 5 min, we observed a hypsochromic shift and reduction in the intensity of the π–π* absorption peak for all six polymer solutions, (Figure 2and Tables 1 and S1), whereas the *n*–π* absorptions remain at approximately the same wavelength and increase in intensity. For many azobenzene chromophores, this behavior is characteristic of isomerization from the *E* to *Z* isomers, for which the π–π* transition band is weaker, but the *n*–π* transition band is stronger.^27,34,35^ Similarly to the absorption spectra of the as-prepared polymers, the π–π* absorption maxima for the irradiated polymers are observed at longer wavelengths (around 305 nm) for polymers **2a**,**b** and **3a**,**b** compared to polymers **1a** and **1b** (around 290 nm).

**Table 1:** λ_max_ Values for the π–π* and *n*–π* Absorptions Observed for As-Prepared and Irradiated Polymers **1**–**3** in Dichloromethane Solution

	as-prepared		irradiated 365 nm		
polymer	λ_max_^π–π*^/nm	λ_max_^*n*-π*^/nm	λ_max_^π–π*^/nm	λ_max_^*n*-π*^/nm	Δλ_max_^π–π*^/nm
**1a**	322	439	296	440	26
**1b**	321	441	290	438	31
**2a**	344	441	303	443	41
**2b**	344	438	304	440	40
**3a**	347	438	307	442	40
**3b**	347	438	307	441	40

Thus, the π–π* absorption maxima for both as-prepared and irradiated polymers **2a**,**b** and **3a**,**b** are bathochromically shifted because of the introduction of the linking group, although the shift is less pronounced for the irradiated polymer solutions. Therefore, the introduction of the linking group increases the separation of the *E* and *Z* π–π* absorption bands.

**Figure 3:**
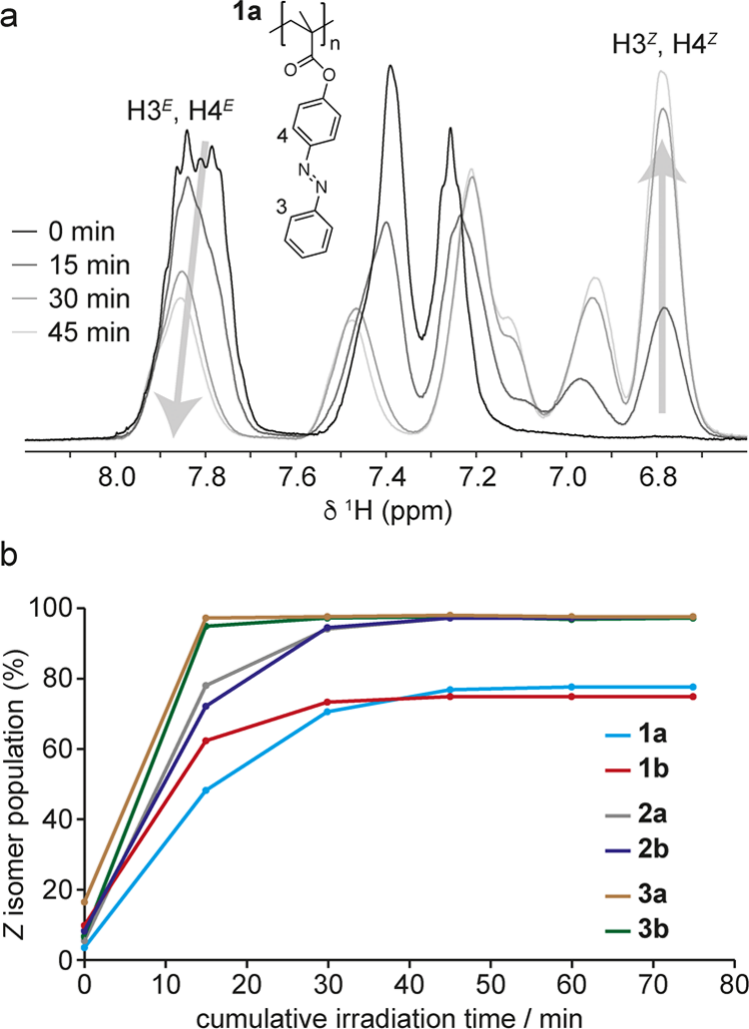
(a) Aromatic regions of ^1^H NMR spectra of **1a** in dichloromethane solution irradiated with 365 nm light for durations between 0 and 105 min. Changes in the intensities of the H3 and H4 resonances for the *E* and *Z* isomeric states are indicated by arrows. (b) Plot of *Z* isomer populations of polymers **1a/b**, **2a/b**, and **3a/b** as a function of cumulative duration of 365 nm irradiation in dichloromethane solution for all polymers studied.

To quantitatively follow the photoisomerization in solution, we recorded ^1^H NMR spectra at 15 min intervals during exposure to 365 nm light (Table S2). Figure 3a shows an example of the changes observed in the NMR spectrum of **1a**. Figure 3b shows the population of *Z* isomers calculated from the NMR spectra as a function of irradiation time for all polymers studied. Polymer solutions **1a** and **1b** reach respective photostationary states (PSSs) consisting of 77 and 75% *Z* isomers after a total irradiation time of approximately 30 min. The PSSs we observe for **1a** and **1b** are comparable to the PSS we measured for monomer **M1a** and to the PSS reported for azobenzene itself when irradiated with 313 nm light.^27^ Therefore, the PSSs for **1a** and **1b** do not appear to be influenced by any steric effects from polymer attachment and are instead a result of the considerable overlap of the π–π* absorption bands of the *E* and *Z* isomers. For azobenzene-based polymer STF materials, this incomplete conversion to the *Z* isomeric state represents a significant limitation on the energy density, since, even under optimum conditions of light absorption, around 20–30% of azobenzene groups remain in the *E* isomeric form, which do not contribute to energy storage and hence impact negatively on the gravimetric energy density.

Figure 3b also shows the growth of the population of *Z* isomers as a function of irradiation time for **2a**/**b** and **3a**/**b**. While the kinetics of photoisomerization are similar to **1a** and **1b**, the *Z* isomer population at the PSS is significantly increased for this set of polymers. In fact, with PSSs of around 98% *Z* isomer, we observed almost quantitative *E* to *Z* conversion, which we attribute to the increased separation of the *E* and *Z* π–π* absorptions resulting from the incorporation of either C_2_H_4_ in **2a**,**b** or the C_4_H_8_ linker in **3a**/**b**. The irradiation wavelength of 365 nm is closer to the shifted absorption maximum for the *E* state, whereas it remains on the edge of the weaker *Z* absorption, thereby significantly reducing the propensity for back conversion to the *E* state during irradiation. This contrasts with polymers **1a** and **1b** where the irradiation wavelength is on the edge of both the *E* and *Z* absorptions, leading to greater back conversion. The increased PSSs for polymers **2a**/**b** and **3a**/**b** mirror observations on similar alkyl-substituted azopolymers,^23^ as well as for fluorine-substituted azobenzene derivatives and arylazopyrazoles, which switch quantitatively between *E* and *Z* isomers due to increased separation of the absorption bands.^28,36^

### Photoisomerization in the Solid State

**Figure 4:**
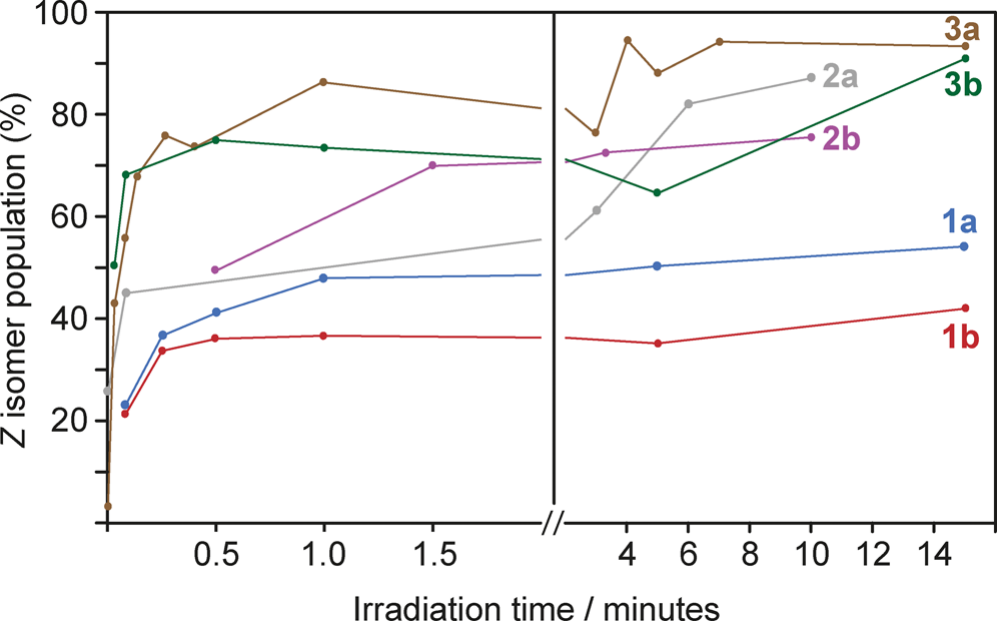
Change of *Z* isomer fractions in the solid-state spin-coated films of polymers **1a/b**, **2a/b**, and **3a/b** as a function of the duration of irradiation with 365 nm light. Each data point corresponds to a unique film.

Films of the as-prepared polymers were prepared by spin-coating at a concentration of 25 g L^–1^ from toluene solution, which produced films of an approximate thickness of around 5 μm (Table S9 and Figure S2). In contrast to films spin-coated from dichloromethane, films spin-coated from toluene had a smooth and homogeneous appearance in optical microscopy and AFM. To investigate the *E* to *Z* photoisomerization in the solid state and quantify the conversion, individual films were irradiated at 365 nm for different lengths of time, dissolved in dichloromethane, and solution ^1^H NMR spectra recorded (Tables S10–15). Figure 4 shows the *Z* isomer population calculated from the NMR spectra as a function of irradiation time for all polymers studied.

The build-up of *Z* isomers happens on a faster timescale than that in solution (c.f. Figure 3), with polymers reaching PSSs within approximately 2 min of irradiation. The precise timescale is likely to be influenced by the light intensity and film thickness; the specific kinetics observed here are unlikely to be a general case. However, the deposition of polymer films does not appear to reduce the timescale of switching, and in this case, appears to hasten it. After 15 min, the solid-state films of **1a** and **1b** show conversion to 56 and 43% *Z* isomer, respectively. The values are significantly reduced compared to the solution-state PSS values of 77 and 75% (vide supra). For **2a** and **2b**, significantly higher PSSs of 85 and 80% *Z* isomer are observed, and for **3a** and **3b**, the solid-state PSS values after 15 min irradiation were found to be 97 and 96% *Z* isomer, respectively, which is equal to the solution state. These results suggest that the inclusion of the alkoxy linker in **2a**/**b** and **3a**/**b** has a marked effect on the PSS achievable under 365 nm irradiation. This may be attributed to the increased π–π* band separation in **2a**/**b** and **3a**/**b**, which leads to better penetration of 365 nm light through the film because far fewer *E* isomers remain in the irradiated portion of the film, and the *Z* isomers that are formed under irradiation do not significantly absorb at 365 nm. However, the reduced *T*_g_ values for the linker-containing polymers may also contribute by imparting more local mobility to the side groups. Indeed, there appears to be a trend of increasing PSS in the solid state with decreasing *T*_g_; which is expected, as a lower *T*_g_ value reflects the increased mobility and free volume a linker of increasing length imparts. Polymers **2b** (*T*_g_ 48 °C, PSS 80%) and **3a** (*T*_g_ 55 °C, PSS 96%) do not follow this trend, which could reflect *T*_g_ being determined by overall chain segment mobility rather than the mobility and free volume of the azobenzene side groups alone. We note that there are significant variations in the measured *Z* isomer populations over the irradiation period, particularly for **3a** and **3b**. For each data point in Figure 4, a separate spin-coated film was studied. However, for each polymer, the spin-coated films were deposited from the same solution. Therefore, we attribute the observed variations in *Z* isomer populations to variations in film thickness between samples and/or inhomogeneities resulting from the spin-coating process. However, the results show that despite these factors, it is possible to achieve high photoconversion for polymers containing linker groups, and for **3a/b**, the photoconversion is not limited by the film thickness.

### Solid-State Thermal Reconversion Kinetics

The required lifetime of the metastable state in an STF material depends upon the specific application; however, lifetimes of at least tens of hours or days would be desirable for applications involving daily repeat energy storage–release cycles. The energy storage lifetime of an STF device can be parameterized by the spontaneous thermal relaxation rate of the metastable state when stored at ambient temperature in the dark. To a first approximation, the thermal relaxation behavior would be expected to follow first-order kinetics described by eq 1
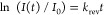
1where *I*_0_ is the initial *Z* isomer population, *I*(*t*) is the *Z* isomer population at time *t*, and *k*_rev_ is the first-order rate constant describing the thermal reconversion process. To quantify the reconversion, irradiated solid-state samples were kept in the dark at room temperature, portions of the sample were taken at specific times and dissolved in dichloromethane, and a solution ^1^H NMR spectrum was recorded (Tables S3–S8).

**Figure 5:**
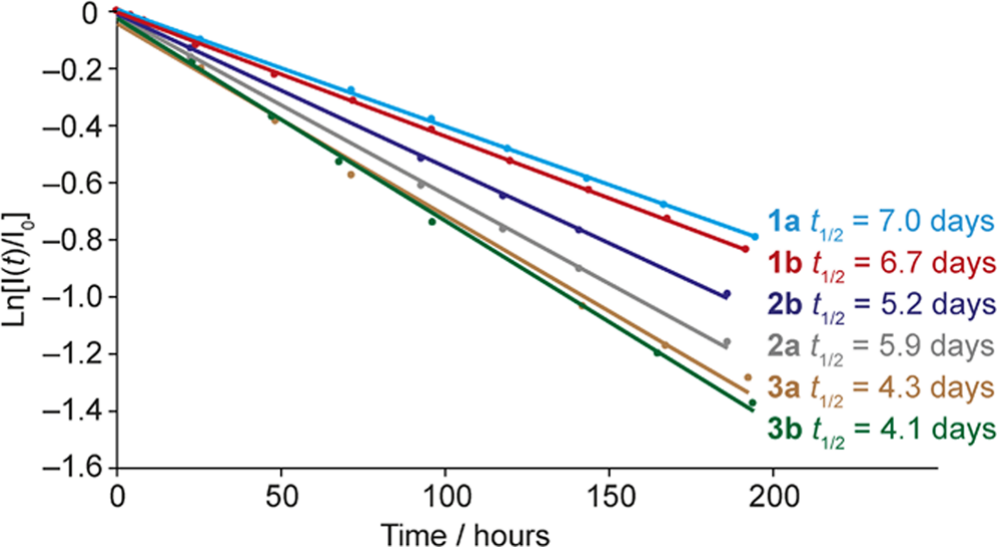
Reconversion kinetics of solid-state samples of **1a/b**, **2a/b**, and **3a/b** stored at ambient temperature in the dark.

Figure 5 shows ln(*I*(*t*)/*I*_0_) measured by ^1^H NMR as a function of time for all polymers studied as well as the corresponding half-lives, *t*_1/2_. Good agreement with first-order kinetics is observed, and polymers **1a** and **1b** show similar half-lives of 7.0 and 6.7 days, respectively. There does not appear to be a correlation between the reconversion kinetics and molecular weight. In the synthesis of this series of polymers, different molecular weight averages were observed for the methacrylates compared to the acrylates, with the methacrylates showing somewhat higher molecular weight averages than the acrylates (see Section 2 in the Supporting Information (SI)). For polymers **1a/b** and **3a/b**, the methacrylates show faster reconversion but for polymers **2a/b**, the acrylate shows faster reconversion, so there is no trend in molecular weight reflected in the reconversion data. However, the introduction of the C_2_H_4_ linker increases the rate of spontaneous thermal reconversion, reducing the half-lives for **2a** and **2b** to 5.2 and 5.9 days, respectively. The introduction of the C_4_H_8_ linker further reduces the half-lives of **3a** and **3b** to 3.6 and 3.3 days, respectively. This suggests that the presence of the linker lowers the activation energy barrier for *Z* to *E* reconversion, and increasing the length of the linker lowers it further. In line with the solid-state irradiation results, it is noteworthy that **2b** (*T*_g_ 48 °C) has a significantly longer half-life than **3a** (*T*_g_ 55 °C), showing that the *T*_g_ of the unirradiated polymers does not directly correlate with the reconversion kinetics. This implies that the backbone mobility of the polymer is not a primary factor in determining the reconversion kinetics, whereas the local mobility and free volume of the azobenzene side group (influenced by the linker length) have the overriding influence.

### Thermal Properties

**Figure 6:**
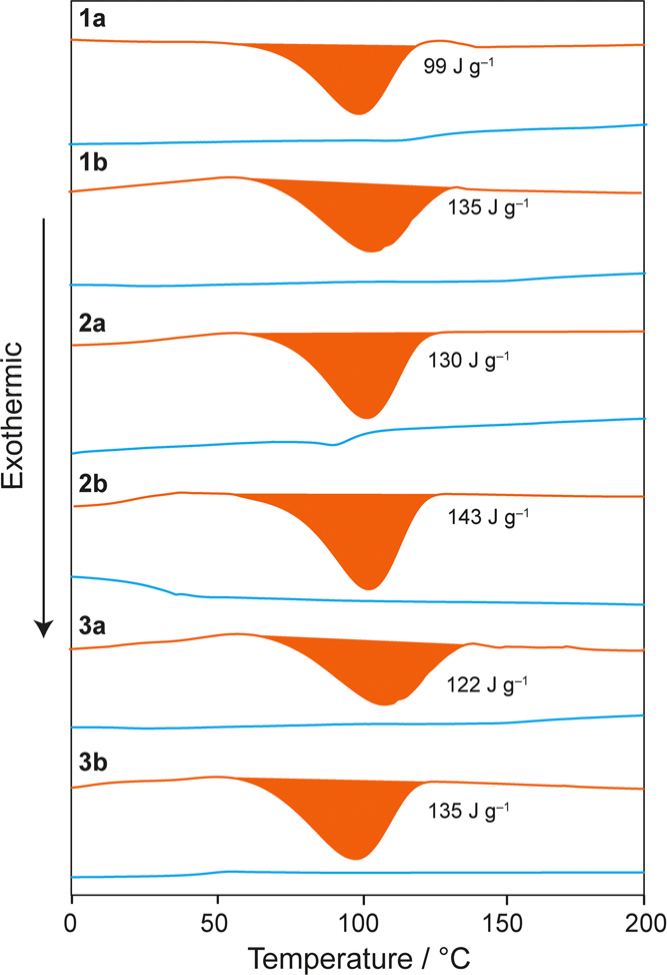
DSC traces for irradiated polymers **1a/b**, **2a/b**, and **3a/b** between 0 and 200 °C recorded with a heating rate of 10 °C min^–1^. Heating curves are shown in orange and cooling curves are shown in blue. Magnitudes of exotherms observed during heating are indicated for each polymer.

We investigated the thermal properties of the six polymers by differential scanning calorimetry (DSC) in the as-prepared state (Figures S4 and S5) and after 365 nm irradiation in solution. *T*_g_ values for the as-prepared samples are reported in Figure 1 and Table 2. For the DSC investigations of irradiated solid-state polymer samples, samples of as-prepared polymers were dissolved in dichloromethane and irradiated with 365 nm light for 60 min to reach the PSS before the solvent was removed under vacuum at ambient temperature in the dark. The resulting solids were transferred to DSC pans for analysis. Figure 6 shows the heating and cooling thermograms of each irradiated polymer sample. On the heating step, broad exothermic thermal features are observed between 60 and 130 °C for each polymer, which we attribute to the thermally driven *Z* to *E* isomerization of the azobenzene side groups.^26^ No exothermic features were observed in subsequent heating steps, although glass transitions were observed at temperatures consistent with unirradiated samples, indicating complete reversion of the side groups to the *E* isomeric state. ^1^H NMR spectra of irradiated samples after the DSC measurement also confirmed that all side groups had reverted to the *E* isomeric state (Figure S6). The enthalpies associated with the observed exotherms for each polymer are summarized in Table 2. Also recorded in Table 2 is the *Z* isomer population in irradiated polymers at the beginning of each DSC measurement, which was separately determined by solution ^1^H NMR on a small fraction of the sample. In general, the *Z* isomer populations in the DSC samples are slightly lower than the PSS values in solution. This is partly attributed to the 1–2 h duration of the drying process, during which some reconversion to the *trans* isomer will have taken place.

**Table 2:** Summary of the Photochemical and Enthalpic Properties of Polymers Studied in This Work

polymer	*T*_g_/°C	normalized energy density^a^/kJ mol^–1^	PSS_365_ (%, solution)	*Z* isomer population at the beginning of DSC measurement (%)	measured energy density/J g^–1^ (DSC)
**1a**	126	41.2 ± 3.6	77 ± 0.2	64	99
**1b**	110	45.9 ± 3.6	75 ± 0.2	73	135
**2a**	87	43.8 ± 1.9	97 ± 0.5	92	130
**2b**	48	46.7 ± 1.5	97 ± 0.5	91	143
**3a**	55	45.1 ± 2.5	98 ± 0.5	88	122
**3b**	28	46.5 ± 1.7	98 ± 0.5	95	135

a Normalized by *Z* isomer population at the beginning of DSC measurement (measured separately by ^1^H NMR).

For **1a**, the gravimetric enthalpy of 99 J g^–1^ we measured in our DSC experiments is in good agreement with the value of 104 J g^–1^ measured by Zhitomirsky et al. Considering the molecular mass of the repeat unit and the *Z* isomer population of 64%, the measured enthalpy corresponds to an isomerization enthalpy of 41.2 kJ mol^–1^. This value is in line with experimental isomerization enthalpies of between 41 and 49 kJ mol^–1^ reported for molecular azobenzene.^37−39^ For **1b**, a higher gravimetric enthalpy of 135 J g^–1^ was measured, which is expected given that the removal of the methyl group in **1b** leads to a reduction in molar mass. This value corresponds to a molar enthalpy of 46.5 kJ mol^–1^. The origin of the difference in molar isomerization enthalpy for **1a** and **1b** is not clear; based on the structures, the isomerization enthalpy of the azobenzene side group would be expected to be comparable in both polymers. While glass transitions for the irradiated polymers were typically not discernible in the DSC traces, we note that the unirradiated form of **1a** shows the highest *T*_g_ of 126 °C, which is at the high-temperature limit of the observed exotherm in the DSC thermogram. If *T*_g_ for irradiated **1a** is lower than this temperature (which would be expected from published data^23^ and due to the increased free volume of *Z* isomer side groups), the glass transition may occur within the exotherm, which could influence its magnitude. In contrast, *T*_g_ values for all other unirradiated polymers are either within or below the temperature range of the exotherm, suggesting that glass transitions for the irradiated forms of these polymers would not coincide with the exotherm.

For **2a** and **2b**, the thermal analysis yields gravimetric enthalpies of 130 and 143 J g^–1^, respectively. These values are significantly higher than that measured for **1a** and comparable to that measured for **1b**, despite the increased mass of the repeat unit due to the presence of the C_2_H_4_ linker group. The reason for this is the higher population of *Z* isomers at the beginning of the experiment (92 and 91%), which means that a larger proportion of the side groups in the sample contribute to the exothermic process. The molar enthalpies of **2a** and **2b** are also very similar to **1b**, confirming that the intrinsic isomerization enthalpy is very similar. The gravimetric enthalpies of **3a** and **3b** (88 and 95% *Z* isomer populations) that contain C_4_H_8_ linkers are 122 and 135 J g^–1^, respectively. These polymers also reach high *Z* isomer populations, and similar molar enthalpies are obtained, but the gravimetric enthalpy is now reduced slightly by the increased mass of the longer C_4_H_8_ linker. ^1^H NMR spectra of the polymers recorded after a full heating–cooling cycle showed an almost complete absence of *Z* isomers, confirming that isomerization of the side groups to the ground-state *E* configuration in these polymers is not limited by any packing effects in the solid state. Instead, the molar enthalpies suggest that the observed thermal energy release is dictated largely by the intrinsic energy difference between the *Z* and *E* isomers.

## Conclusions

This work shows that the STF properties of polymers with azobenzene side groups can be significantly enhanced through a judicious choice of structural units. Characterization of the basic acrylate and methacrylate polymers with directly attached azobenzene side groups shows that the photoswitching properties mirror those of molecular azobenzene, which has a photostationary state of below 80% Z isomer under 365 nm irradiation. The molar energy density during thermally driven reconversion to the ground-state *E* isomer agrees with the reconversion enthalpy for molecular azobenzene, showing that inclusion within the polymer does not significantly affect the photoswitching properties. Separation of the azobenzene moieties from the polymer backbone via an alkoxy linker group separates the π–π* absorption bands of the *E* and *Z* isomers, leading to almost quantitative photoisomerization. Although the molar *Z* → *E* reconversion enthalpy remains similar to that of molecular azobenzene, the increased PSS significantly increases the gravimetric energy density by up to 44% compared to polymers with directly attached side groups. Experiments on polymer films suggest that the increased absorption band separation may also give a greater light penetration depth, which would be beneficial for energy storage in macroscopic films and coatings. The inclusion of the alkoxy linkers also reduces the glass transition temperatures of the polymers, which leads to somewhat faster spontaneous thermal reconversion; however, half-lives of the *Z* isomeric states remain more than 4 days, providing scope for applications with storage–release cycles on timescales of up to several days.

## Materials and Methods

### Materials and Reagents

All reagents and solvents used in the synthesis of the monomers were readily available commercially and used as supplied without further purification. Reactions were monitored by thin-layer chromatography (TLC) using Merck silica gel 60 F254 plates (0.25mm). TLC plates were visualized using UV light (254nm) and/or by using the appropriate TLC stain. Flash column chromatography was performed using silica gel (VWR) 40–63 μm in combination with a solvent specified in the procedure (see the Supporting Information). Reactions under anhydrous and inert conditions were conducted in oven-dried glassware under an atmosphere of argon. Triple detection gel permeation chromatography (GPC) was carried out using a Shimadzu RID-20A, with both Wyatt Technologies miniDAWN Treos and Wyatt Technologies Viscostar II viscometer detectors. The mobile phase was HPLC-grade tetrahydrofuran. Samples were run on a Phenomenex Penogel 5 μm linear (2) column in conjunction with a guard. All data obtained from chromatographic traces were analyzed by ASTRA 6 software from Wyatt Technology. The molar mass distribution of a particular polymer was determined from the retention volume of the chromatographic peak maximum and the retention volume range of the peak. Samples were prepared in the mobile phase at a concentration of 1 mg mL^–1^. UV–vis absorption measurements were carried out using a Cary 60, in a 1 cm pathlength quartz cuvette. The UV–vis spectra were collected between 200 and 800 nm at 200 nm min^–1^ using Cary WinUV software. Samples were prepared in dichloromethane, unless stated otherwise, to a concentration of around 3.91 × 10^–3^ g L^–1^. Precise concentrations were obtained by weighing the sample on a Mettler Toledo XPE205 DeltaRange balance and diluting using volumetric flasks with a resulting error of ±0.015 × 10^–4^ g L^–1^. Sample irradiations were carried out with an OmniCure LX500 Ultra-compact UV LED spot curing system. Irradiation was performed at 10 mm from the 365 nm UV LED spot curing head equipped with a 12 mm focusing lens. Optical microscopy was performed on a Zeiss Axio Scope.A1 microscope and used in conjunction with a Canon 700D digital camera. Scanning electron microscopy was performed on a JEOL JSM 7800F, with samples mounted on ITO-coated glass slides. Any errors reported are the standard deviation of repeat experiments unless otherwise stated; in such circumstances, the error relates to the systematic error inherent to the performed experimental method where error analysis has been undertaken.

## Supporting Information Available

Materials and methods; synthetic procedures; and additional characterization details and data (PDF)
