# Supplementary material for: Understanding Solid-State Photochemical Energy Storage in Polymers with Azobenzene Side Groups
Source: ACS Appl Mater Interfaces. 2023 Jun 23;15(26):31787–94. doi: 10.1021/acsami.3c04631 (PMC10326853; doi:10.1021/acsami.3c04631)
Supplement: Supplementary file 1 — am3c04631_si_001.pdf [file am3c04631_si_001.pdf]

Supporting Information

Understanding Solid-state Photochemical Energy Storage in Polymers with Azobenzene Side Groups

Callum Wallace,[1] Kieran Griffiths,[1] Benjamin L. Dale,[1] Stuart Roberts,[2] Jonathan Parsons,[2] John M. Griffin,[1,3]* Verena Görtz[1]*

[1]*Department of Chemistry, Lancaster University, Lancaster, LA1 4YB, United Kingdom*

[2]*Jaguar Land Rover Research, International Digital Laboratory, University of Warwick, CV4 7AL, United Kingdom*

[3]*Materials Science Institute, Lancaster University, Lancaster LA1 4YB, United Kingdom*

**Corresponding author. Email: v.gortz@lancaster.ac.uk, j.griffin@lancaster.ac.uk*

Table of Contents

1. Materials and methods

2. Synthetic procedures

3. UV-vis absorption measurements

4. Photostationary state determination in solution

5. Determination of *Z* isomer reconversion kinetics in the solid state

6. Photostationary state determination in spin-coated films

7. Differential scanning calorimetry

8. Compound Characterization Spectra

9. References

1. Materials and methods

All reagents and solvents used in the synthesis of the monomers were readily available commercially and used as supplied without further purification. Reactions were monitored by thin-layer chromatography (TLC) using Merck silica gel 60 F254 plates (0.25mm). TLC plates were visualized using UV light (254nm) and/or by using the appropriate TLC stain (Cerium Molybdate stain). Flash column chromatography was performed using silica gel (VWR) 40-63 μm in collaboration with a solvent system specified in the procedure. Solvents were removed by rotary evaporator in vacuo at 40 °C and the compounds further dried using high vacuum lines. Reactions under anhydrous and inert conditions were conducted in oven-dried glassware under an inert atmosphere of argon.

Infrared spectra were recorded using an Agilent Technologies Cary 630 FT-IR Spectrometer and analysed using Agilent MicroLab PC software. Reported absorptions are either strong or medium strength unless otherwise stated and given in wavenumbers (cm^{-1}). Spectra were recorded of azobenzene-based polymers and monomers in the form of solid powders.

Melting points were obtained *via* differential scanning calorimetry (DSC).

^{1}H and ^{13}C solution state Nuclear Magnetic Resonance spectra were recorded using a Bruker Fourier 400 NMR spectrometer at 400 MHz and 100 MHz, respectively, at 298K. Chemical shifts (δ) are quoted downfield from tetramethylsilane (TMS), referenced to solvent signals: ^{1}H δ = 7.27 ($CHCl_3$), 5.55 (CH_2Cl_2), ^{13}C δ = 77.0 ($CDCl_3$). NMR spectra were viewed and analysed using TopSpin NMR software.

The molar mass of all monomers synthesised was confirmed *via* mass spectrometry. The mass spectroscopy data was recorded using a Shimadzu LCMS-IT-TOF in electrospray ionisation (ESI) or atmospheric pressure chemical ionisation (APCI) mode.

Molar mass distributions of all polymers synthesised were determined by triple detection Gel Permeation Chromatography (GPC). The GPC data was collected on a Shimadzu GPC/SEC set up using a Shimadzu RID-20A, with both a Wyatt Technologies miniDAWN Treos and a Wyatt Technologies Viscostar II viscometer detectors. The mobile phase was HPLC grade tetrahydrofuran. Samples were run on a Phenomenex Penogel 5μ Linear (2) column in conjunction with a guard. All data obtained from chromatographic traces were analysed by ASTRA 6 software from Wyatt technology. The molar mass distribution of a particular polymer was determined from the retention volume of the chromatographic peak maximum and the retention volume range of the peak, respectively. Samples were prepared in the mobile phase of concentration 1 mg mL^{-1}.

UV-vis absorption measurements were carried out using a Cary 60, in a 1 cm pathlength quartz cuvette. The UV-vis spectra were collected between 200 nm – 800 nm at 200 nm min^{-1} using Cary WinUV software. Samples were prepared in dichloromethane, unless stated otherwise, to a concentration around 3.91×10^{-3} g L^{-1}. Precise concentrations were obtained by weighing the sample on a Mettler Toledo XPE205 DeltaRange balance and diluting using volumetric flasks with a resulting error of $\pm 0.015 \times 10^{-4}$ g L^{-1}.

Sample irradiations were carried out with an OmniCure® LX500 Ultra-compact UV LED spot curing system. Irradiation was performed at 10 mm from the 365 nm UV LED spot curing head equipped with a 12 mm focusing lens.

Optical microscopy was performed on a Zeiss Axio Scope.A1 microscope and used in conjunction with a Canon 700D digital camera.

Scanning electron microscopy was performed on a JEOL JSM 7800F, with samples mounted on ITO coated glass slides. Refractive index values were measured using an ABBE 5 refractometer alongside an acetone standard. Measurements were made for azobenzene-based polymers films coated using a deposition concentration of 25 mg mL^{-1}. Preliminary contact profilometry surface data were recorded using an SJ-400 surface profilometer. Measurements were made for azobenzene-based polymers films coated using a deposition concentration of 25 mg mL-1.

Any errors reported are the standard deviation of repeat experiments unless otherwise stated, in such circumstance the error relates to the systematic error inherent to the preformed experimental method where error analysis has been undertaken.

2. Synthetic procedures

2.1 Monomer synthesis

4-Methacryloyloxyazobenzene (M1a)[1]

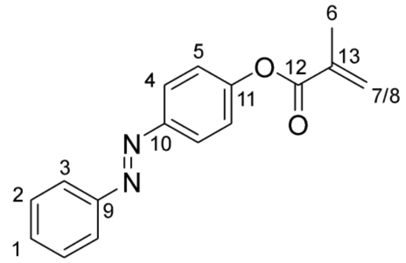

A solution of 4-phenylazophenol (4.4 g, 22.0 mmol) was dissolved in 50 mL of tetrahydrofuran, to which triethylamine (3.1 mL, 22.2 mmol) and 2,6-di-*tert*-butyl-*p*-cresol (2.0 mg) were added. The solution was purged with argon gas and stirred for 30 min at room temperature. Methacryloyl chloride (6.4 mL, 66.0 mmol) was added dropwise while the solution temperature was kept below 5 °C by using an ice bath. The reaction flask was left stirring for 48 hrs at room temperature. The resulting precipitate was filtered, and the solution was diluted four times with 3:1 mix of dichloromethane (CH_2Cl_2, 150 mL) and water (50 mL). The organic phase was then washed and extracted with saturated sodium bicarbonate (100 mL), 1M sodium hydroxide (NaOH, 100 mL), 0.5M sodium hydroxide (100 mL), 1M hydrochloric acid (HCl, 100 mL), and saturated brine solution (100 mL). The organic phase was dried with sodium sulfate (Na_2SO_4) and the residual solvent was removed in *vacuo*. The resultant material was purified by flash chromatography using 1:1 CH_2Cl_2: petroleum ether 40 – 60 and dried under high vacuum overnight. $R_f = 0.33$

Yield: orange powder, 3.6 g, 61%

^{1}H NMR (400 MHz, $CDCl_3$) δ_H(ppm): 8.00 (d, 2H, J=9.0 Hz, H^4), 7.96 - 7.92 (m, 2H, H^3), 7.57 - 7.47 (m, 3H, H^1 and H^2), 7.32 (d, 2H, J=8.9 Hz, H^5), 6.42 (t, 1H, J=1.1 Hz, H^8 *cis*), 5.82 (q, 1H, J=1.5 Hz, H^7 *trans*), 2.11 (dd, 3H, J=1.10, 1.5 Hz, H^6).

^{13}C NMR (100 MHz, $CDCl_3$) δ_C(ppm): 165.5 (C^{12}); 152.9 (C^{11}); 152.6 (C^9); 150.2 (C^{10}); 135.7 (C^{13}); 131.0 (C^1); 129.1 (C^2); 127.6 ($C^{7/8}$); 124.0 (C^5); 122.8 (C^3); 122.2 (C^4); 18.4 (C^6).

FT-IR ν_{max} (cm^{-1}): 3449 (C-H), 2922 (C-H), 1731 (C=O), 1638 (C=C), 1589 (C=C).

ES-MS (m/z): $C_{16}H_{14}N_2O_2$ calculated $[M^{+}H]^{+}$ *m/z* 267.1128, measured, 267.1115.

Mp: 111 °C – 116 °C

4-Acryloyloxyazobenzene (M1b)[2,3]

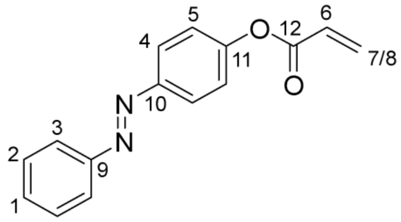

A solution of 4-phenylazophenol (4.5 g, 23.0 mmol) was dissolved in 50 mL of tetrahydrofuran (THF), to which triethylamine (TEA) (3.1mL, 22.2 mmol) and 2,6-di-*tert*-butyl-*p*-cresol (2.0 mg) were added. The solution was purged with argon gas and stirred for 30 min at room temperature. Acryloyl chloride (5.0 g, 4.5 mL, 55.0 mmol) was added dropwise, while the solution temperature was kept below 5 °C by using an ice bath. The reaction flask was left stirring for 70 hrs at room temperature. The resulting precipitate was filtered, and the solution was diluted four times with 3:1 mix of dichloromethane (CH_2Cl_2, 150 mL) and water (50 mL). The organic phase was then washed and extracted with saturated sodium bicarbonate (100 mL), 1M sodium hydroxide (NaOH, 100 mL), 0.5M sodium hydroxide (100 mL), 1M hydrochloric acid (HCl, 100 mL), and saturated brine solution (100 mL). The organic phase was dried with sodium sulfate (Na_2SO_4) and the residual solvent was removed in *vacuo*. The resultant material was purified by flash chromatography using 2:1 CH_2Cl_2: petroleum ether 40 – 60. The product was dried under high vacuum overnight. R_f = 0.27

Yield. orange solid, 3.8 g, 65.5%.

^{1}H NMR (400 MHz, $CDCl_3$) δ_H(ppm): 8.00 (d, 2H, J=8.9 Hz, H^4), 7.97 - 7.91 (m, 2H, H^3), 7.58 - 7.47 (m, 3H H^1 and H^2), 7.33 (d, 2H, J=9.0 Hz, H^5), 6.67 (dd, 1H, J=17.3, 1.2 Hz, H^7 *cis*), 6.38 (dd, 1H, J=17.4, 10.4 Hz, H^6 *trans*), 6.08 (dd, 1H, J=10.5, 1.2 Hz, H^8).

^{13}C NMR (100MHz, $CDCl_3$) δ_C(ppm): 164.2 (C^{12}); 152.6 (C^9 and C^{11}); 150.3 (C^{10}); 133 ($C^{7/8}$); 131.1 (C^1); 129.1 (C^2); 127.7 (C^6); 124.1 (C^5); 122.9 (C^3); 122.2 (C^4).

FT-IR ν_{max} (cm^{-1}): 3457 (C-H), 3058 (C-H), 1735 (C=O), 1636 (C=C), 1586 (C=C).

ES-MS (m/z): $C_{15}H_{12}N_2O_2$ calculated $[M^{+}H]^{+}$ *m/z* 253.0972, measured, 253.0966.

Mp: 64 °C – 68 °C

^{1}H NMR (400 MHz, $CDCl_3$) δ_H(ppm): 6.04 (dd, 1H, J=1.9, 1.0 Hz, H^7 *Cis*), 5.50 (dd, 1H, J=1.9, 1.7 Hz, H^8 *Trans*), 4.13 (t, 2H, J=6.5 Hz, H^5), 3.62 (t, 2H, J=6.4 Hz, H^2), 2.81 (b, 1H, H^1), 1.88 (dd, 1H, J=1.7, 1.0 Hz, H^6), 1.77 – 1.66 (m. 2H, H^4), 1.66 (m, 2H, H^3).

FT-IR ν_{max} (cm^{-1}): 3412 (OH), 2946 (C-H), 2950 (C-H), 1714 (C=O), 1636 (C=C).

This compound has previously been synthesised and reported.[330]

4-(Phenyldiazenyl)phenoxyethyl methacrylate (M2a)[4]

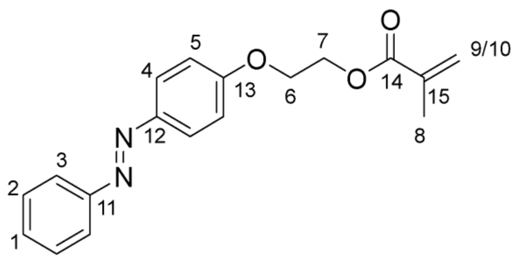

A solution of 4-phenylazophenol (2.4 g, 12 mmol) and triphenylphosphine (5.25 g, 20 mmol) was dissolved in 100 mL of dichloromethane (CH_2Cl_2) and purged with argon gas. Under inert conditions 4-hydroxyethylmethacrylate (2.0 mL, 16 mmol) was added dropwise to the reaction mixture and the resulting solution was cooled to 0 °C. Over a 30-minute period diisopropyl azodicarboxylate (DIAD) (3.9 mL, 20.0 mmol) was added dropwise to the solution, while maintaining a temperature below 5 °C. The reaction was left stirring overnight for 68 hours at room temperature. The resultant material was purified by flash chromatography using dichloromethane (CH_2Cl_2) as the eluting solvent. R_f = 0.45

Yield. orange crystals, 3.06 g, 9.87 mmol, 83%.

^{1}H NMR (400 MHz, $CDCl_3$) δ_H(ppm): 7.92 (d, 2H, J=9.0 Hz, H^4), 7.90 - 7.85 (m, 2H, H^3), 7.56 - 7.42 (m, 3H, H^2 and H^1), 7.06 (d, 2H, J=9.0 Hz, H^5), 6.19 - 6.18 (m, 1H, H^9 *trans*), 5.63 - 5.63 (m, 1H, H^{10} *cis*), 4.58 - 4.55 (t, 2H, J = 4.8 Hz, H^6), 4.35 - 4.33 (t, 2H, J = 4.8 Hz, H^7), 1.99 (dd, 3H, J = 1.6, 1.0 Hz, H^8).

^{13}C NMR (100MHz, $CDCl_3$) δ_C(ppm): 167.3 (C^{16}); 161.0 (C^{15}); 152.7 (C^{13}); 147.3 (C^{14}); 135.9 (C^1); 130.5 ($C^{9/10}$); 129.1 (C^2); 126.2 (C^{15}); 124.8 (C^4); 122.6 (C^3); 114.9 (C^5); 66.2 (C^7); 62.9 (C^6); 18.3 (C^8).

FT-IR ν_{max} (cm^{-1}): 3040 (C-H), 2963 (C-H), 1705 (C=O), 1631 (C=C), 1601(C=C).

APCI-MS (m/z): $C_{18}H_{18}N_2O_3$ calculated [M$^+$Na]$^+$ *m/z* 311.1392, measured, 311.1396.

Mp: 70 - 72 °C

^{1}H NMR (400 MHz, $CDCl_3$) δ_H(ppm): 8.08 - 7.96 (m, 4H, H^3 and H^4), 7.56 - 7.49 (m, 3H, H^1 and H^2), 7.02 - 6.93 (d, 2H, H^5), 6.22 - 6.17 (m, 1H, H^{10}), 5.87 - 5.81 (m, 1H, H^9), 4.62 - 4.49 (m, 2H, H^6), 4.36 - 4.27 (m, 2H, H^7), 1.95 - 1.89 (m, 3H, H^8).

^{13}C NMR (100MHz, $CDCl_3$) δ_C(ppm): 165.8, 161.3, 153.5, 144.7, 136.9, 131.0, 128.2, 123.3, 122.8, 115.1, 73.5, 67.6, 18.2.

4-(Phenyldiazenyl)phenoxyethyl acrylate (M2b)

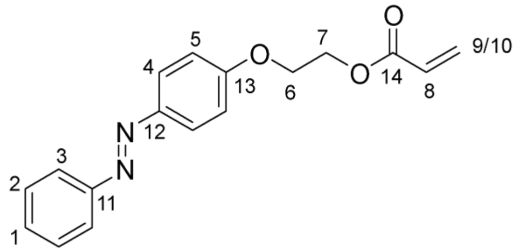

A solution of 4-phenylazophenol (2.4 g, 12 mmol) and triphenylphosphine (5.25 g, 20 mmol) was dissolved in 100 mL of dichloromethane (CH_2Cl_2) and purged with argon gas. Under inert conditions 4-hydroxyethyl acrylate (1.8 mL, 16 mmol) was added dropwise to the reaction mixture and the resulting solution was cooled to 0 °C. Over a 30-minute period diisopropyl azodicarboxylate (DIAD) (3.9 mL, 20.0 mmol) was added dropwise to the solution, while maintaining a temperature below 5 °C. The reaction was left stirring overnight for 68 hours at room temperature. The resultant material was purified by flash chromatography using dichloromethane (CH_2Cl_2) as the eluting solvent. R_f = 0.38

Yield. orange crystals, 1 g, 3.4 mmol, 28%.

^{1}H NMR (400 MHz, $CDCl_3$) δ_H(ppm): 7.92 (d, 2H, J=9.0 Hz, H^4), 7.90 - 7.85 (m, 2H, H^3), 7.52 - 7.50 (m, 2H, H^2), 7.49 - 7.43 (m, 1H, H^1), 7.04 (d, 2H, J=9.1 Hz, H^5), 6.49 - 6.45 (dd, 1H, J=17.3, 1.4 Hz, H^{10} *trans*), 6.22 – 6.15 (dd, 1H, J=17.3, 10.4 Hz, H^8), 5.89 – 5.86 (dd, 1H, J=10.4, 1.4 Hz, H^9 *cis*), 4.57 – 4.55 (t, 2H, J=4.8 Hz, H^6), 4.32 – 4.30 (t, 2H, J=4.8 Hz, H^7).

^{13}C NMR (100MHz, $CDCl_3$) δ_C(ppm): 166.1 (C^{14}); 160.9 (C^{13}); 152.7 (C^{11}); 147.3 (C^{12}); 131.5 (C^1); 130.5 (C^8); 129.1(C^2); 128.0 ($C^{9/10}$); 124.8 (C^4); 122.6 (C^3); 114.8 (C^5); 66.2 (C^7); 62.7 (C^6).

FT-IR ν_{max} (cm^{-1}): 3424 (C-H), 2958 (C-H), 1716 (C=O), 1638 (C=C), 1597(C=C).

APCI-MS (m/z): $C_{17}H_{16}N_2O_3$ calculated $[M^{+}Na]^{+}$ *m/z* 297.1225, measured, 297.1222.

Mp: 62 °C

4-Hydroxybutyl acrylate

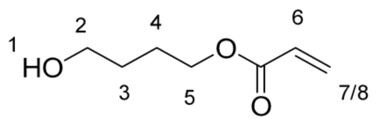

A solution of 1,4-butanediol (6.9 mL, 78 mmol), EDAC (5.0 g, 26 mmol) and DMAP (0.67 g, 5.5 mmol) was dissolved in 110 mL of dichloromethane (CH_2Cl_2). acrylic acid (2.2 mL, 26 mmol) was added to the vessel dropwise and the reaction left to stir for 14 hours overnight at room temperature. The resultant material was purified by flash chromatography using 1:1 hexane: ethyl acetate. R_f = 0.38

Yield. clear oil, 2.2 g, 14.1 mmol, 54.2%.

4-(4-Phenylazophenoxy)butyl methacrylate (M3a)[5]

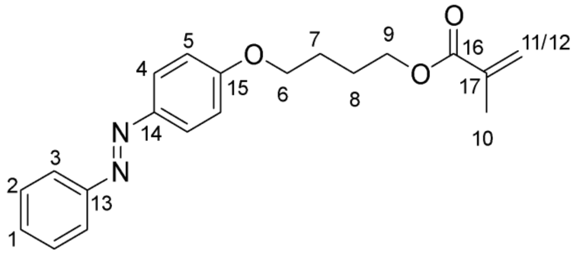

A solution of 4-phenylazophenol (2.4 g, 12 mmol) and triphenylphosphine (5.25 g, 20 mmol) was dissolved in 100 mL of dichloromethane (CH_2Cl_2) and purged with argon gas. Under inert conditions 4-hydroxybutylmethacrylate (2.1 g, 13 mmol) was added dropwise to the reaction mixture and the resulting solution was cooled to 0 °C. Over a 30-minute period diisopropyl azodicarboxylate (DIAD) (3.9 mL, 20.0 mmol) was added dropwise to the solution, while maintaining a temperature below 5 °C. The reaction was left stirring overnight for 14 hours at room temperature. The resultant material was purified by flash chromatography using 2:1 dichloromethane (CH_2Cl_2): petroleum ether 40 – 60. R_f = 0.43

Yield. orange crystals, 3.4 g, 10.0 mmol, 83.7%.

^{1}H NMR (400 MHz, $CDCl_3$) δ_H(ppm): 7.94 (d, 2H, J=9.0 Hz, H^3), 7.91 - 7.88 (m, 2H, H^4), 7.55 - 7.50 (m, 2H, H^2), 7.48 - 7.43 (m, 1H, H^1), 7.03 (d, 2H, J=9.0 Hz, H^5), 6.15 - 6.13 (m, 1H, H^{11} *trans*), 5.60 – 5.58 (m, 1H, H^{12} *cis*), 4.27 (t, 2H, J=6.0 Hz, H^9), 4.12 (t, 2H, J=5.9 Hz, H^6), 1.98 (dd, 3H, J=1.58, 1.0 Hz, H^{10}), 1.97 - 1.86 (m, 4H, H^7 and H^8).

^{13}C NMR (100MHz, $CDCl_3$) δ_C(ppm): 167.5 (C^{16}); 161.4 (C^{15}); 152.8 (C^{13}); 147.0 (C^{14}); 136.4 (C^{17}); 130.4 (C^1); 129.1(C^4); 125.4 ($C^{11/12}$); 124.8 (C^5); 122.6 (C^2); 114.8 (C^3); 67.6 (C^6); 64.2 (C^9); 25.9 (C^7); 25.4 (C^8); 18.4 (C^{10})

FT-IR ν_{max} (cm^{-1}): 3436 (C-H), 2931 (C-H), 1707 (C=O), 1636 (C=C), 1582(C=C).

APCI-MS (m/z): $C_{20}H_{22}N_2O_3$ calculated $[M^+H]^+$ *m/z* 339.1703, measured, 339.1703

Mp: 80 °C – 82 °C

4-(4-Phenylazophenoxy)butyl acrylate (M3b)[6]

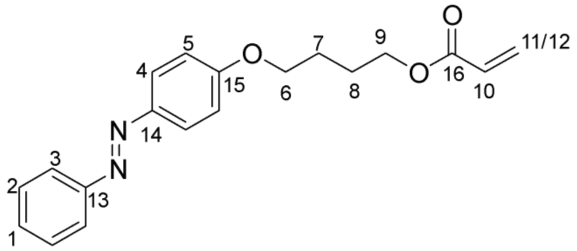

A solution of 4-phenylazophenol (2.4 g, 12 mmol) and triphenylphosphine (5.25 g, 20 mmol) was dissolved in 100 mL of dichloromethane (CH_2Cl_2) and purged with argon gas. Under inert conditions 4-hydroxybutyl acrylate (2.22 mL, 16 mmol) was added dropwise to the reaction mixture and the resulting solution was cooled to 0 °C. Over a 30-minute period diisopropyl azodicarboxylate (DIAD) (3.9 mL, 20.0 mmol) was added dropwise to the solution, while maintaining a temperature below 5 °C. The reaction was left stirring overnight for 14 hours at room temperature. The resultant material was purified by flash chromatography using 2:1 dichloromethane (CH_2Cl_2): petroleum ether 40 – 60 and recrystalised from petroleum ether 40 – 60 (5.0 ml). R_f = 0.42

Yield. orange crystals, 2.4 g, 7.4 mmol, 61.7%.

^{1}H NMR (400 MHz, $CDCl_3$) δ_H(ppm): 8.00 (d, 2H, J=9.0 Hz, H^4), 7.91 - 7.87 (m, 2H, H^3), 7.56 - 7.49 (m, 2H,H^2), 7.48 - 7.42 (m, 1H, H^1), 7.02 (d, 2H, J=9.1 Hz, H^5), 6.43 (dd, 1H, J= 17.4, 1.5 Hz H^{12} *cis*), 6.15 (dd, 1H, J= 17.0, 10.5 Hz, H^{10}), 5.85 (dd, 1H, J=10.5, 1.5 Hz, H^{11} *trans*), 4.29 (t, 2H, J=6.1 Hz, H^9), 4.11 (t, 2H, J=5.9 Hz, H^6), 2.00 - 1.88 (m, 4H, H^7 and H^8).

^{13}C NMR (100MHz, $CDCl_3$) δ_C(ppm): 166.2 (C^{16}); 161.4 (C^{15}); 152.7 (C^{13}); 147.0 C^{14}); 130.7 (C^1); 130.3 ($C^{11/12}$); 129.0 (C^2); 128.4 (C^{10}); 124.7 (C^4); 122.5 (C^3); 114.6 (C^5); 67.6 (C^6); 64.1 (C^9); 25.8 (C^7); 25.4 (C^8)

FT-IR ν_{max} (cm^{-1}): 3438 (C-H), 2950 (C-H), 1723 (C=O), 1600 (C=C), 1580(C=C).

APCI-MS (m/z): $C_{19}H_{20}N_2O_3$ calculated $[M^{+}Na]^{+}$ *m/z* 325.1547, measured, 325.1533.

Mp: 68 °C – 70 °C

2.2 Polymer synthesis

General method:

In a typical polymerisation reaction, the monomer (1.1 mmol) was dissolved in 3.0 mL of anhydrous tetrahydrofuran (THF), and azobisisobutyronitrile (AIBN) (9 mg, 5.5 *x* 10^{-2} mmol) was added. The solution was subjected to 3 freeze/pump/thaw cycles. The pump segments were maintained for a minimum of 2 minutes and the monomer mixture was thawed under argon. The reaction was left under inert conditions at 65°C for 3 days. The resulting polymer was precipitated methanol (100 mL) under vigorous stirring and collected by filtration.

Poly(4-methacryloyloxyazobenzene) (1a)[7]

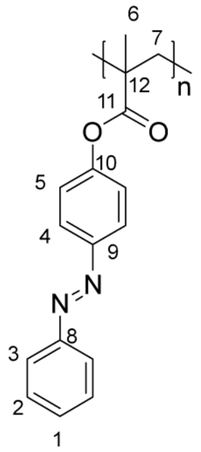

Yield. orange powder, 0.2 g, 70.3%

^{1}H NMR (400 MHz, $CDCl_3$) δ_H(pmm): 8.00 - 7.69 (b, 4H, H^3 and H^4), 7.52 - 7.32 (b, 3H, H^1 and H^2), 7.27 - 7.14 (b, 2H, H^5), 2.75 - 1.89 (b, m, 2H, H^7), 1.78 - 1.15 (b, m, 3H, H^6)

^{13}C NMR (100 MHz, $CDCl_3$) δ_C(pmm): 175.2 (C^{11}); 152.6 (C^{10}); 152.4 (C^8); 150.4 (C^9); 131.1 (C^1); 129.0 (C^2); 124.2 (C^3); 123.0 (C^4); 122.6 (C^5); 46.0 (C^{12}); 26.1 (C^7); 25.1 (C^6).

GPC: M_n (THF): 7.2 *x* 10^3 $gmol^{-1}$

Polydispersity (M_w/M_n) = 1.7.

DSC: T_g = 126 °C (10 °C min^{-1}).

FT-IR ν_{max} (cm^{-1}): 3060 (C-H), 2987 (C-H), 1746 (CO), 1591 (C=C).

Poly(4-acryloyloxyazobenzene) (1b)[8]

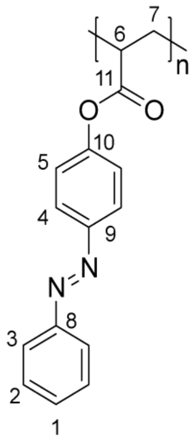

Yield. light orange powder, 0.16 g, 57.7%

^1H NMR (400 MHz, $CDCl_3$) δ_H(pmm): 7.98 - 7.68 (b, 4H, H^3 and H^4), 7.56 - 7.33 (b, 3H, H^1 and H^2), 7.31 - 7.10 (b, 2H, H^5), 3.25 – 2.97 (b, 1H, H^6) 2.97 - 1.22 (b, m, 2H, H^7).

^{13}C NMR (100MHz, $CDCl_3$) δ_C(pmm): 175.2 (C^{11}); 152.4 (C^{10}); 152.2 (C^8); 150.5 (C^9); 131.0 (C^1); 129.0 (C^2); 124.2 (C^3); 123.0 (C^4); 122.2 (C^5); 41.8 (C^6); 26.9 (C^7).

GPC: M_n (THF): 4.5 *x* 10^3 $gmol^{-1}$

Polydispersity (M_w/M_n) = 1.2.

DSC: T_g = 116 °C (10 °C min^{-1}).

FT-IR ν_{max} (cm^{-1}): 3062 (C-H), 2926 (C-H), 1744 (C=O), 1591 (C=C).

Poly(4-(phenyldiazenyl)phenoxyethyl methacrylate) (2a)

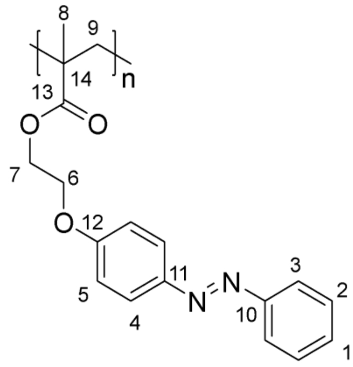

Yield. light orange powder, 0.35 g, 73%

^1H NMR (400 MHz, $CDCl_3$) δ_H(pmm): 7.93 - 7.70 (b, 4H, H^3 and H^4), 7.54 - 7.29 (b, 3H, H^1 and H^2), 7.11 – 6.77 (b, 2H, H^5), 4.40-3.79 (b, 4H, H^6 and H^7), 2.25 – 1.63 (b, m, 2H, H^9), 1.63 – 0.65 (b, 3H, H^8).

^{13}C NMR (100MHz, $CDCl_3$) δ_C(pmm): 177.2 (C^{13}); 160.8 (C^{12}); 152.5 (C^{10}); 147.0 (C^{11}); 130.4 (C^1); 129.0 (C^2); 124.6 (C^4); 122.5 (C^3); 114.7 (C^5); 65.4 (C^6); 63.1 (C^7); 50.1 (C^{14}); 46.8 (C^9); 44.7 (C^8).

GPC: M_n (THF): 1.39 *x* 10^4 $gmol^{-1}$

Polydispersity (M_w/M_n) = 2.34

DSC: T_g = 88 °C (10 °C min^{-1}).

FT-IR ν_{max} (cm^{-1}): 3062 (C-H), 2939 (C-H), 1729 (C=O), 1599 (C=C), 1582 (C=C).

Poly(4-(phenyldiazenyl)phenoxyethyl acrylate) (2b)

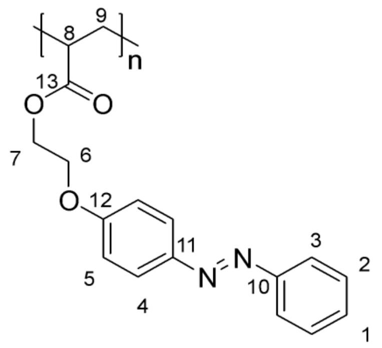

Yield. light orange powder, 0.22 g, 22%

^{1}H NMR (400 MHz, $CDCl_3$) δ_H(pmm): 7.95 - 7.71 (b, 4H, H^3 and H^4), 7.55 - 7.32 (b, 3H, H^1 and H^2), 7.05 – 6.83 (b, 2H, H^5), 4.59-3.84 (b, 4H, H^6 and H^7), 2.76 – 1.08 (b, m, 3H, H^8 and H^9).

^{13}C NMR (100MHz, $CDCl_3$) δ_C(pmm): 174.2 (C^{13}); 160.9 (C^{12}); 152.6 (C^{10}); 147.0 (C^{11}); 130.4 (C^1); 129.0 (C^2); 124.7 (C^4); 122.5 (C^3); 114.8 (C^5); 67.6 (C^6); 64.3 (C^7); 41.1 (C^8); 29.6 (C^9).

GPC: M_n (THF): 2.3 x 10^3 $gmol^{-1}$

Polydispersity (M_w/M_n) = 1.59.

DSC: T_g = 46 °C (10 °C min^{-1}).

FT-IR ν_{max} (cm^{-1}): 3066 (C-H), 2924 (C-H), 1731 (C=O), 1597 (C=C), 1582 (C=C).

Poly(4-(4-phenylazophenoxy)butyl methacrylate) (3a)[5]

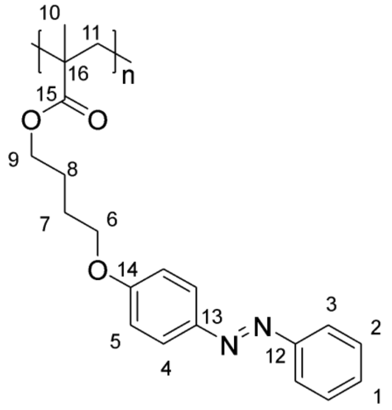

Yield. light orange powder, 0.86 g, 78.5%

^{1}H NMR (400 MHz, $CDCl_3$) δ_H(pmm): 7.94 - 7.73 (b, 4H, H^3 and H^4), 7.54 - 7.33 (b, 3H, H^1 and H^2), 7.03 – 6.82 (b, 2H, H^5), 4.19-3.85 (b, 4H, H^6 and H^9), 2.20 – 0.75 (b, m, 9H, H^7, H^8, H^{11} and H^{10}).

^{13}C NMR (100MHz, $CDCl_3$) δ_C(pmm): 177.4 (C^{15}); 161.3 (C^{14}); 152.7 (C^{12}); 146.9 (C^{13}); 130.4 (C^1); 129.0 (C^2); 124.8 (C^4); 122.6 (C^3); 114.6 (C^5); 67.6 (C^6 and C^9); 64.8 (C^7 and C^8); 45.1 (C^{16}); 25.5 (C^{10} and C^{11}).

GPC: M_n (THF): 1.1 x 10^4 $gmol^{-1}$

Polydispersity (M_w/M_n) = 1.88

DSC: T_g = 55 °C (10 °C min^{-1}).

FT-IR ν_{max} (cm^{-1}): 3063 (C-H), 2948 (C-H), 2873 (C-H), 1727 (C=O), 1599 (C=C).

Poly(4-(4-Phenylazo phenoxy)butyl acrylate) (3b)

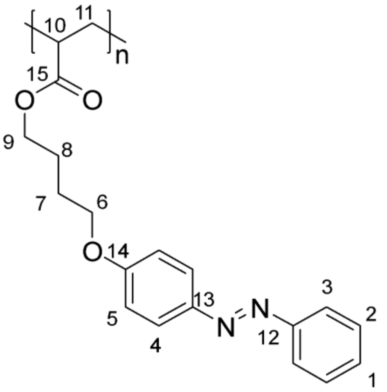

Yield. orange powder, 0.13 g, 36.8%

^1H NMR (400 MHz, $CDCl_3$) δ_H(pmm): 7.95 - 7.72 (b, 4H, H^3 and H^4), 7.54 - 7.33 (b, 3H, H^1 and H^2), 7.04 – 6.84 (b, 2H, H^5), 4.34-3.69 (b, 4H, H^6 and H^9), 2.72 - 1.12 (b, m, 7H, H^7, H^8, H^{10} and H^{11}).

^{13}C NMR (100MHz, $CDCl_3$) δ_C(pmm): 174.5 (C^{15}); 161.4 (C^{14}); 152.7 (C^{12}); 147.0 (C^{13}); 130.4 (C^1); 129.0 (C^2); 124.8 (C^4); 122.6 (C^3); 114.7 (C^5); 67.6 (C^6 and C^9); 64.3 (C^7 and C^8); 41.6 (C^{10}); 25.7 (C^{11}).

GPC: M_n (THF): 4.5 *x* 10^3 $gmol^{-1}$

Polydispersity (M_w/M_n) = 1.21.

DSC: T_g = 28 °C (10 °C min^{-1}).

FT-IR ν_{max} (cm^{-1}): 3060 (C-H), 2961 (C-H), 2871 (C-H), 1725 (C=O), 1597 (C=C).

3. UV-vis absorption measurements

UV-vis absorption measurements were carried out using a Cary 60, in a 1 cm pathlength quartz cuvette. The UV-vis spectra were collected between 200 nm – 800 nm at 200 nm min^{-1} using Cary WinUV software. Samples were prepared in dichloromethane, unless stated otherwise, to a concentration around 3.91 *x* 10^{-3} g L^{-1}. Precise concentrations were obtained by weighing the sample on a Mettler Toledo XPE205 DeltaRange balance and diluting using volumetric flasks with a resulting error of ±0.015 *x* 10^{-4} g L^{-1}.

Molar extinction coefficients of all irradiated and unirradiated monomers and polymers synthesised were calculated from the respective UV-vis spectrum of known concentrations following the Beer-Lambert Law. The error reported in these values reflects the systematic error in accurately determining the concentration of the samples.

UV-vis absorption spectra of irradiated samples were obtained from samples irradiated in solution for periods of 5 minutes. The light source was set to 50% intensity; therefore, the light intensity used was approximately 0.025 W cm^{-1}. The cuvettes were sealed with PTFE stoppers to prevent solvent evaporation during irradiation. The cuvettes and samples were shielded from external light sources during exposure, transit, and measurement.

Table S1. The absorption maxima and extinction coefficients of π→π* and n→π* transitions recorded for monomer **M1a** and polymers **1** - **3** in dichloromethane. Values for irradiated samples acquired after exposure to 365 nm light for a duration of 5 minutes at which point no further changes were observed in the absorption spectra with continued irradiation.

Sample	π→π* λ_{max} (nm)	π→π* ε (L cm^{-1} mol^{-1})	n→π* λ_{max} (nm)	n→π* ε (L cm^{-1} mol^{-1})
**M1a**	325	1.85 *x* 10^4	442	6.43 *x* 10^2
**M1a** irradiated	294	1.17 *x* 10^4	436	1.58 *x* 10^3
**1a**	322	1.57 *x* 10^4	439	4.93 *x* 10^2
**1a** irradiated	296	9.44 *x* 10^3	440	1.02 *x* 10^3
**1b**	321	2.30 *x* 10^4	441	6.90 *x* 10^2
**1b** irradiated	290	7.23 *x* 10^3	438	1.47 *x* 10^3
**2a**	344	2.29 *x* 10^4	441	9.87 *x* 10^2
**2a** irradiated	303	7.00 *x* 10^3	443	2.04 *x* 10^3
**2b**	344	2.90 *x* 10^4	438	1.19 *x* 10^3
**2b** irradiated	304	9.06 *x* 10^3	440	2.63 *x* 10^3
**3a**	347	2.63 *x* 10^4	438	1.79 *x* 10^3
**3a** irradiated	307	8.67 *x* 10^3	442	2.59 *x* 10^3
**3b**	347	2.10 *x* 10^4	436	1.03 *x* 10^3
**3b** irradiated	307	7.94 *x* 10^3	441	2.08 *x* 10^3

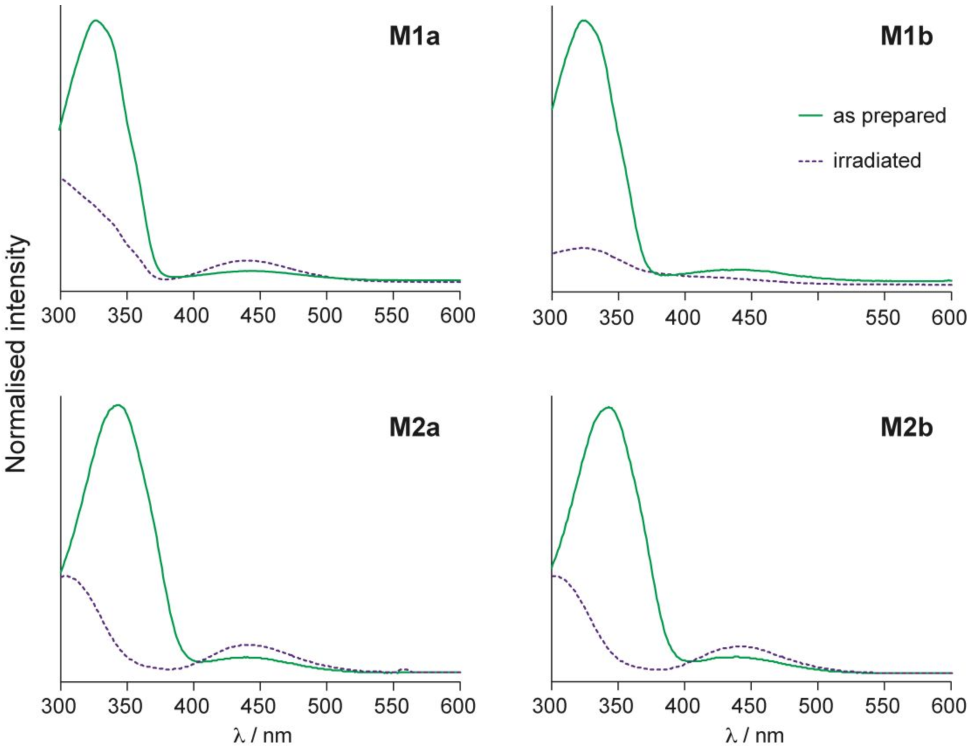

Figure S1. UV-vis absorption spectra for monomers **M1a/b** and **M2a/b**. All spectra were recorded in CH_2Cl_2. Spectra for each monomer are normalised to the maximum intensity of the as prepared monomer.

4. Photostationary state determination in solution

Each sample (15 mg) was dissolved in dichloromethane (0.8 mL) within an NMR tube. The NMR tube was fitted with a J Young NMR valve. The tube was sealed and irradiated with NMR spectra collected at 15-minute intervals. The population of *Z* isomers was determined *via* integration of the *E* and *Z* resonances. Irradiation repeated until the ratio between the *E* and *Z* integrations was seen to plateau. Z isomer populations at each irradiation interval for each sample are shown in Table S2.

Table S2. *Z* isomer populations measured by ^{1}H NMR spectroscopy during irradiation in dichloromethane solution with 365 nm light.

	Z isomer population (%)					
Irradiation time (minutes)	1a	1b	2a	2b	3a	3b
0	3.4	9.6	5.0	8.0	16.2	6.5
15	47.9	62.0	77.7	71.7	96.9	94.5
30	70.3	72.9	93.5	94.1	97.3	96.7
45	76.5	74.4	96.6	96.8	97.4	97.1
60	77.3	74.4	96.9	96.9	97.1	96.3

75	77.3	74.5	96.9	97.1	97.2	96.6

5. Determination of *Z* isomer reconversion kinetics in the solid state

The thermal reconversion rates of samples were determined using the following procedure. The polymer material (50 mg) was dissolved in dichloromethane (3.5 mL) within a borosilicate glass vial. The vial was sealed and placed 10 cm from the UV focusing lens in a dark room. The vial was then exposed to UV light at 365 nm for 120 minutes. The vial was wrapped in aluminium foil and the irradiated solution dried under reduced pressure at ambient temperature. The resulting residue was stored in the dark and a sample was taken at specific times, dissolved in dichloromethane, and a solution ^{1}H NMR spectrum recorded. The Z isomer population at each time interval was determined by integration of the *E* and *Z* isomer resonances. Tables S3 – S8 show *Z* isomer populations at time intervals following irradiation.

Table S3. *Z* isomer population following irradiation for **1a**.

**1a**	
Time after irradiation (hours)	*Z* isomer population (%)
0.0	75.4
25.5	68.2
71.6	57.1
95.9	51.5
119.3	46.5
143.5	41.9
166.9	38.2
194.8	34.1

Table S4. *Z* isomer population following irradiation for **1b**.

**1b**	
Time after irradiation (hours)	*Z* isomer population (%)
0	72.5
1	72.1
4	71.4
8	70.1

24	64.4
48	57.9
72	52.8
96	47.8
120	42.8
144	38.7
168	35.0
192	31.4

Table S5. *Z* isomer population following irradiation for **2a**.

**2a**	
Time after irradiation (hours)	*Z* isomer population (%)
0.0	96.5
22.6	82.1
92.7	52.3
117.9	44.9
141.0	39.1
186.3	30.3
257.3	21.2
280.6	18.4
305.5	16.3
329.2	15.1

Table S6. *Z* isomer population following irradiation for **2b**.

**2b**	
Time after irradiation (hours)	*Z* isomer population (%)
0.0	96.9
22.6	84.8
92.7	57.8

117.9	50.6
141.0	45.0
186.3	35.9
257.3	26.1
280.6	23.7
305.5	21.3
329.2	19.5

Table S7. *Z* isomer population following irradiation for **3a**.

**3a**	
Time after irradiation (hours)	*Z* isomer population (%)
0.0	96.4
25.7	78.7
48.4	65.5
71.5	54.3
142.1	34.2
167.5	29.9
192.7	26.6

Table S8. *Z* isomer population following irradiation for **3b**.

**3b**	
Time after irradiation (hours)	*Z* isomer population (%)
0.0	96.7
22.9	80.7
47.4	66.6
67.9	57.0
96.2	46.1
164.9	29.2
194.1	24.4

6. Photostationary state determination in spin-coated films

Polymer films were prepared on quartz substrates by spin coating from toluene solutions at a concentration of 25 g L^{-1}. Spin coating was achieved by a 4-step procedure as shown in Table S9. This procedure was experimentally optimised to give the flattest and most homogenous films, although it was not possible to avoid a thicker rim remaining at the edge of the substrate. Toluene was used as the casting solvent because this produced the most homogenous films, as shown in Figure S2.

Table S9. Spin coating procedure for preparation of polymer films.

Stage	Rotation rate (rpm)	Acceleration (rpm s^{-1})	Duration (s)	Purpose
1	120	60	20	Initial rotation to spread polymer solution over substrate
2	500	1000	30	Spin coating step
3	1000	5000	20	Removal of excess solution from film edges
4	190	300	90	Film drying

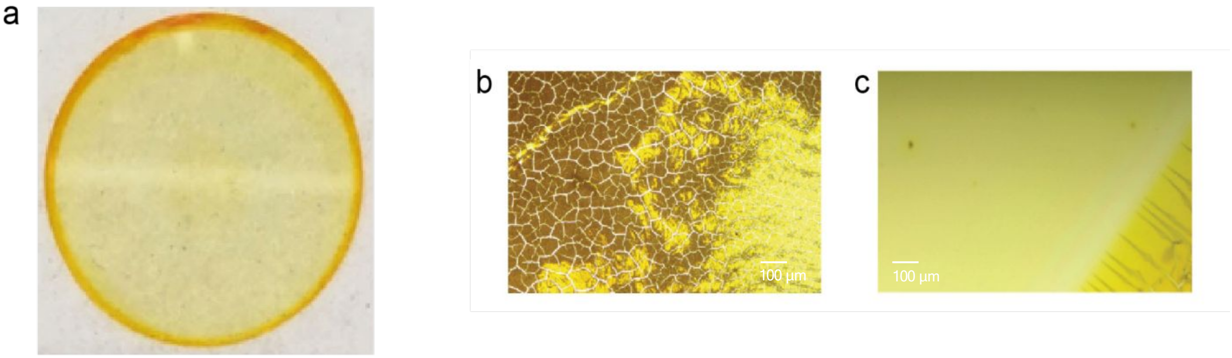

Figure S2. (a) Photograph of a glass disc spin-coated with polymer **1a** using the procedure given in Table S9. (b,c) Photomicrographs of spin-coated films of polymer **1a** deposited from (b) dichloromethane and (c) toluene at 100 times magnification.

The film thickness was estimated using scanning electron microscopy (SEM). A coated disc was snapped in half and mounted vertically in the SEM so that the thickness could be measured. This method only provides an estimation of the film thickness because it is difficult to determine the precise orientation of the sample, meaning that the apparent film thickness can be affected by parallax error. To mitigate this, defects and cracks at the film edge were used to help ensure the sample was perpendicular to the plane of the image, as shown in Figure S3.

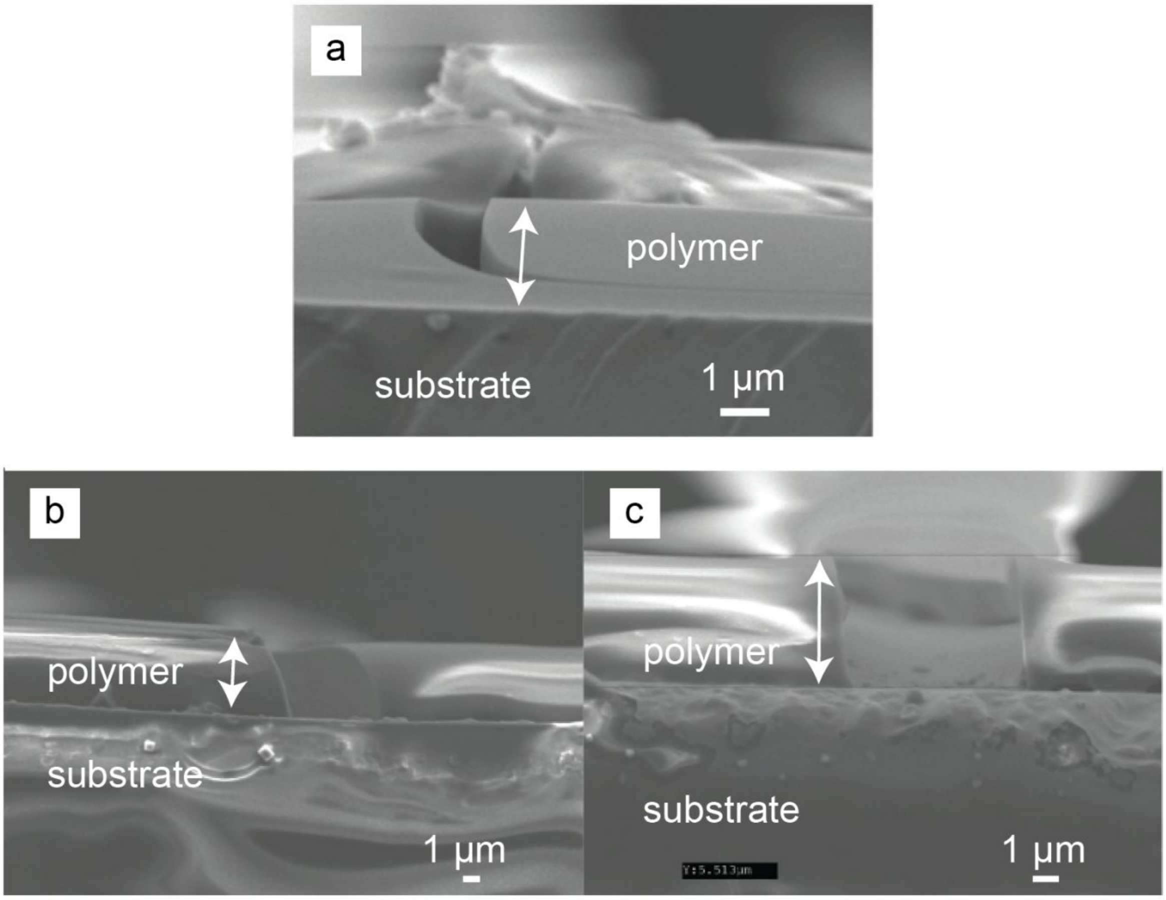

Figure S3. SEM images of polymer films on glass substrates. Panels (a, b, c) show views from different angles and locations.

The *E*-*Z* photoisomerisation kinetics of spin-coated films were investigated by irradiation spin-coated 1 inch glass coverslips using the 365 nm LED setup described in Section 1. Separate spin coated films were irradiated for different periods of time before the film was dissolved in 0.7 ml dichloromethane and the ^{1}H NMR spectrum was recorded. The *Z* isomer population in each film for each irradiation duration is shown in Table S10.

Table S10. *Z* isomer populations as a function of irradiation duration for spin-coated films of **1a**.

Irradiation duration (sec)	*Z* isomer population (%)
5	22.9
15	36.6
30	41.0
60	47.8
300	50.3
900	54.1

1800	56.2
2700	55.6

Table S11. *Z* isomer populations as a function of irradiation duration for spin-coated films of **1b**.

Irradiation duration (sec)	*Z* isomer population (%)
5	21.2
15	33.6
30	36.0
60	36.5
300	35.2
900	41.9
1800	43.8
2700	43.3

Table S12. *Z* isomer populations as a function of irradiation duration for spin-coated films of **2a**.

Irradiation duration (sec)	*Z* isomer population (%)
5	44.9
180	60.8
360	81.7
600	86.8

Table S13. *Z* isomer populations as a function of irradiation duration for spin-coated films of **2b**.

Irradiation duration (sec)	*Z* isomer population (%)
5	49.2
30	69.6
90	72.2
200	75.2
600	79.3

Table S14. *Z* isomer populations as a function of irradiation duration for spin-coated films of **3a**.

Irradiation duration (sec)	*Z* isomer population (%)
2	42.6
5	55.4
8	67.5
16	75.5
24	73.0
60	86.1
60	85.8
180	75.7
240	94.2
300	87.6
420	94.0
900	93.0

Table S15. *Z* isomer populations as a function of irradiation duration for spin-coated films of **3b**.

Irradiation duration (sec)	*Z* isomer population (%)
2	50.0
5	67.8
30	74.7
60	73.2
300	64.4
900	90.7
1800	95.4
2700	95.4

7. Differential scanning calorimetry

Thermal analysis was performed on a Mettler Toledo DSC 1 with STARe acquisition and analysis software calibrated against an indium standard. DSC measurements were

carried out on material samples between 2 mg - 8 mg. Unless otherwise stated all samples were analysed between -30 °C and 200 °C at a ramp rate of 10 °C min^{-1} under nitrogen.

To determine the glass transition temperatures, samples of the as-prepared polymers were heated to 170 °C within the DSC, and allowed to cool in the furnace. The samples were then subjected to a heat/cool cycle at 10 °C min^{-1} and the glass transition temperatures determined from the second heat. DSC data for all polymers is shown in Figures S4 and S5.

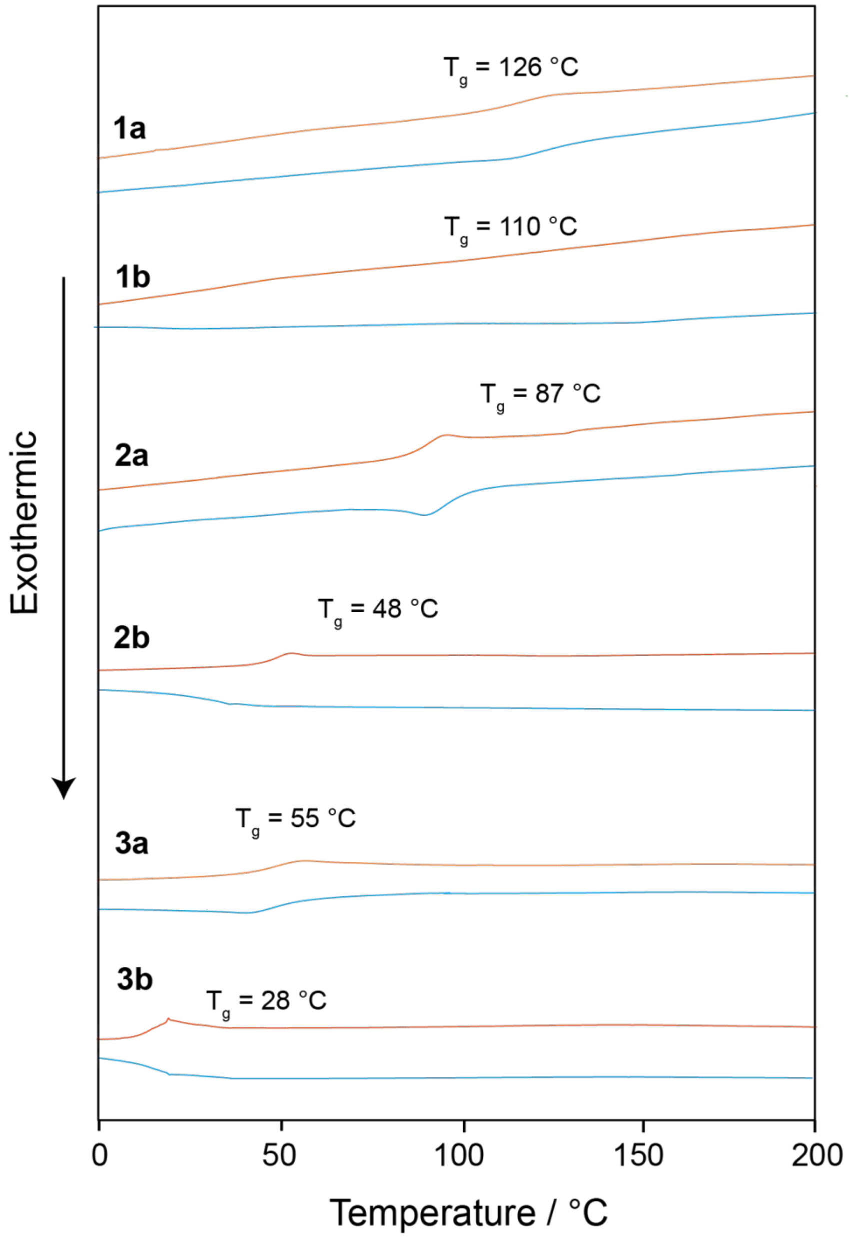

Figure S4. DSC traces for as-prepared polymers between 0 - 200 °C. Heating curves are shown in orange and cooling curves are shown in blue.

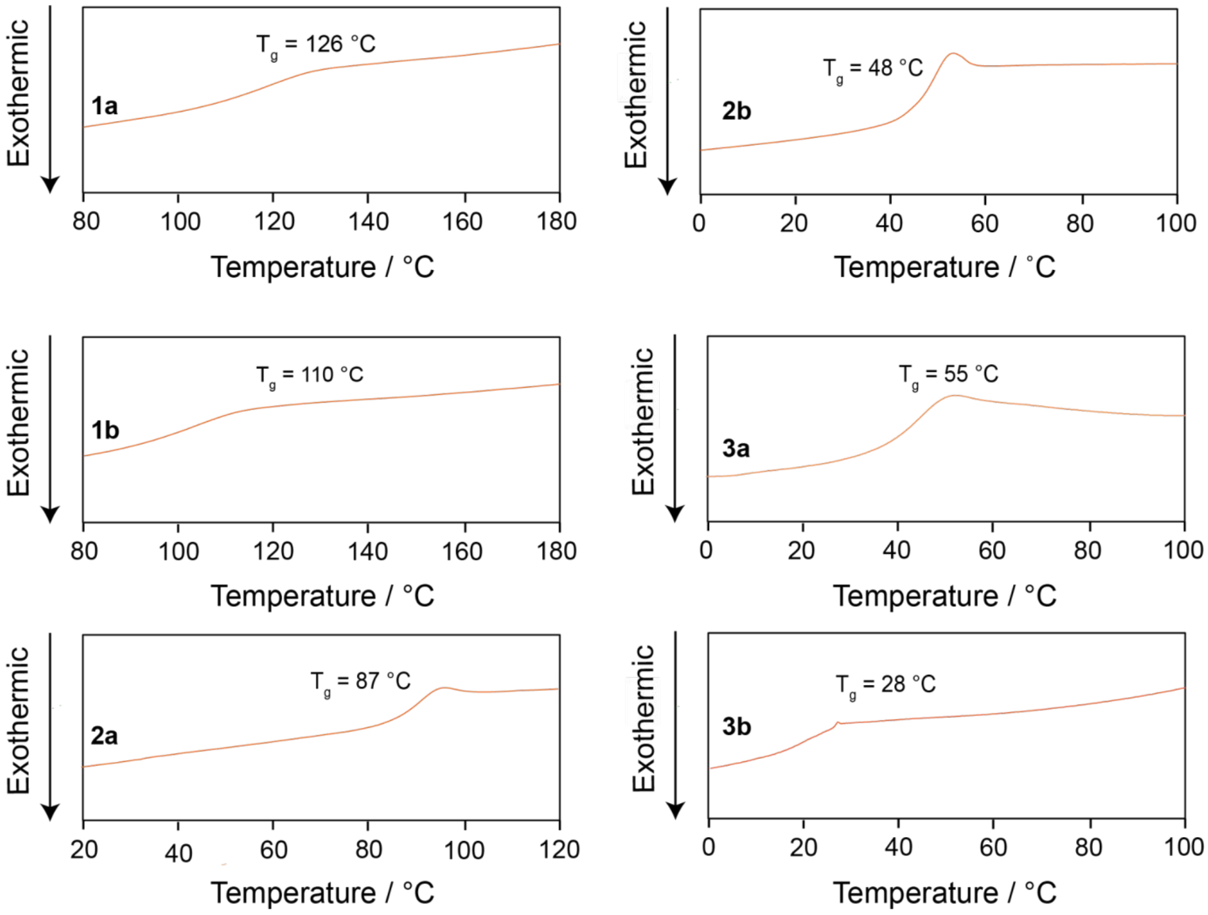

Figure S5. Expanded views of the heating curves in Figure 7.1 showing glass transitions.

Irradiated samples for thermal analysis were prepared using solution irradiation according to the following procedure. A polymer sample (10 mg) was dissolved in dichloromethane (5 g L^{-1}). The sample vial was sealed and held 10 cm from the UV focusing lenses in a dark room. The vial was then exposed to UV light at 365 nm for 60 minutes. After UV exposure the solvent was removed in vacuo in the dark and the samples were then subjected to a heat/cool cycle at 10°C min^{-1} and the values for the exotherm determined from the first heat. ^{1}H NMR spectra of samples in the unirradiated and irradiated states, and immediately following a DSC measurement are shown in Figure S6.

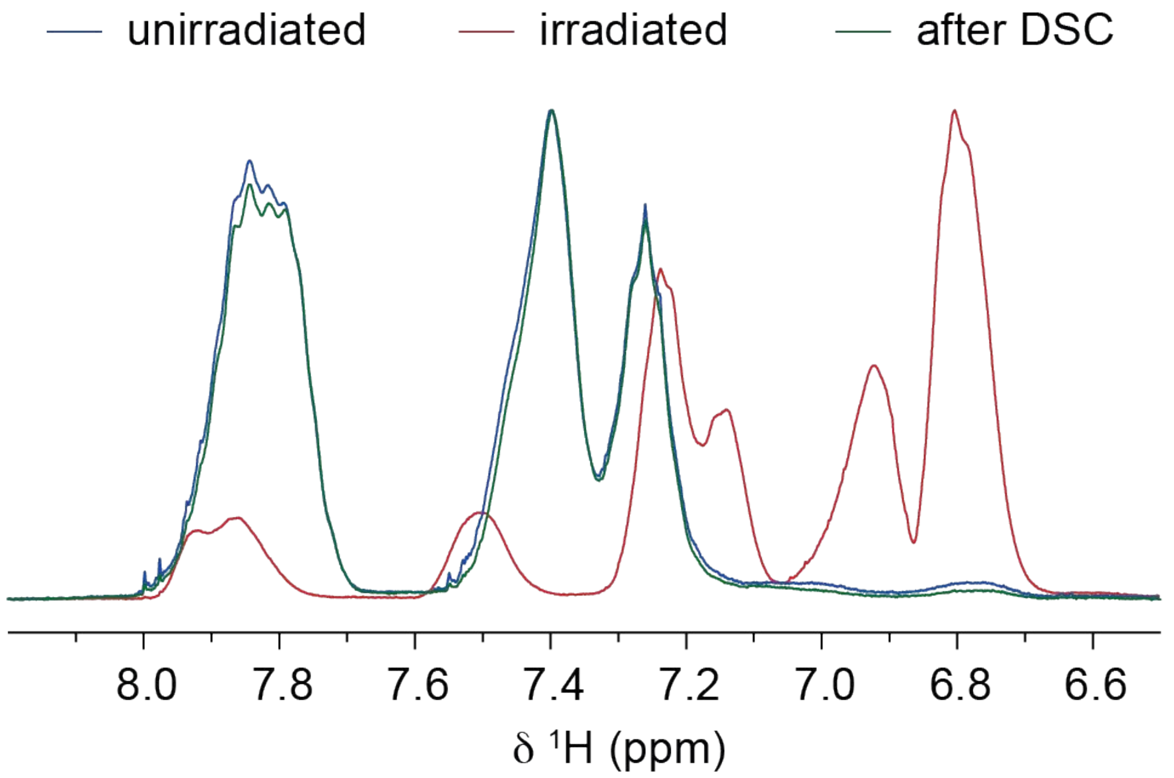

Figure S6. ^{1}H NMR spectra of **1a** in the unirradiated and irradiated states, and following a DSC measurement. Each sample was loaded into a DSC pan following the procedure outlined in the main text, before being dissolved in CH_2Cl_2 for the NMR measurement.

8. Compound Characterization Spectra

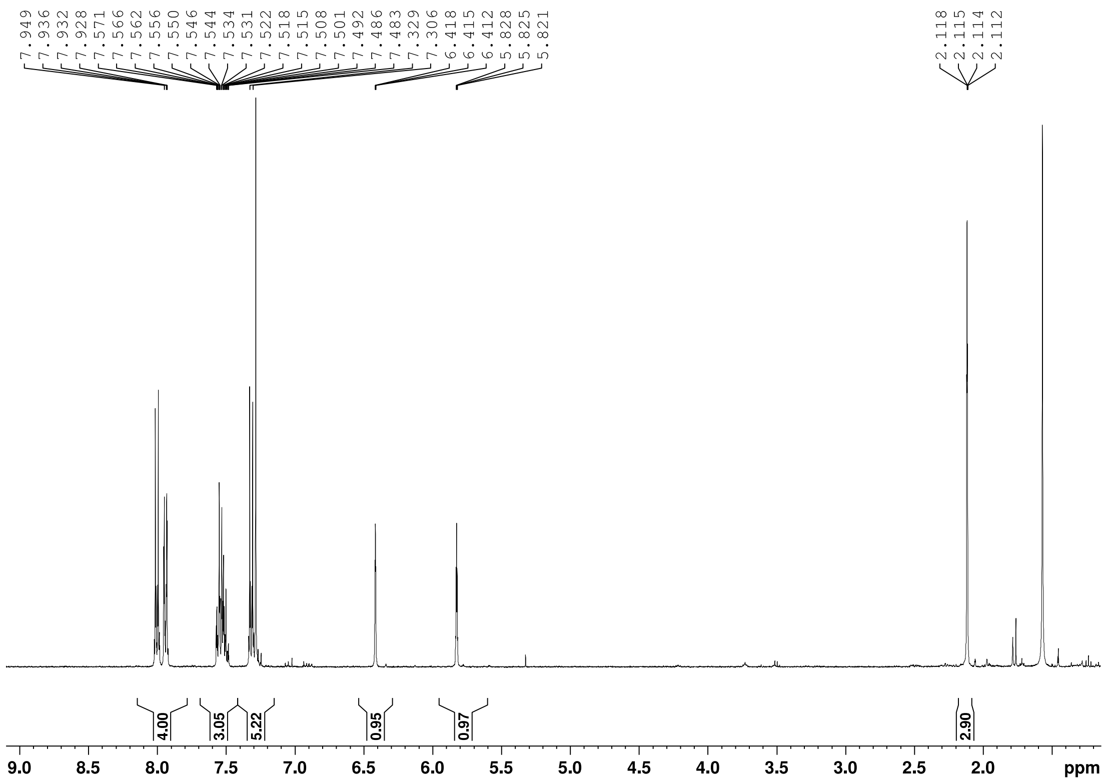

Figure S7. 400 MHz ($CDCl_3$)^{1}H NMR spectrum of monomer **M1a**.

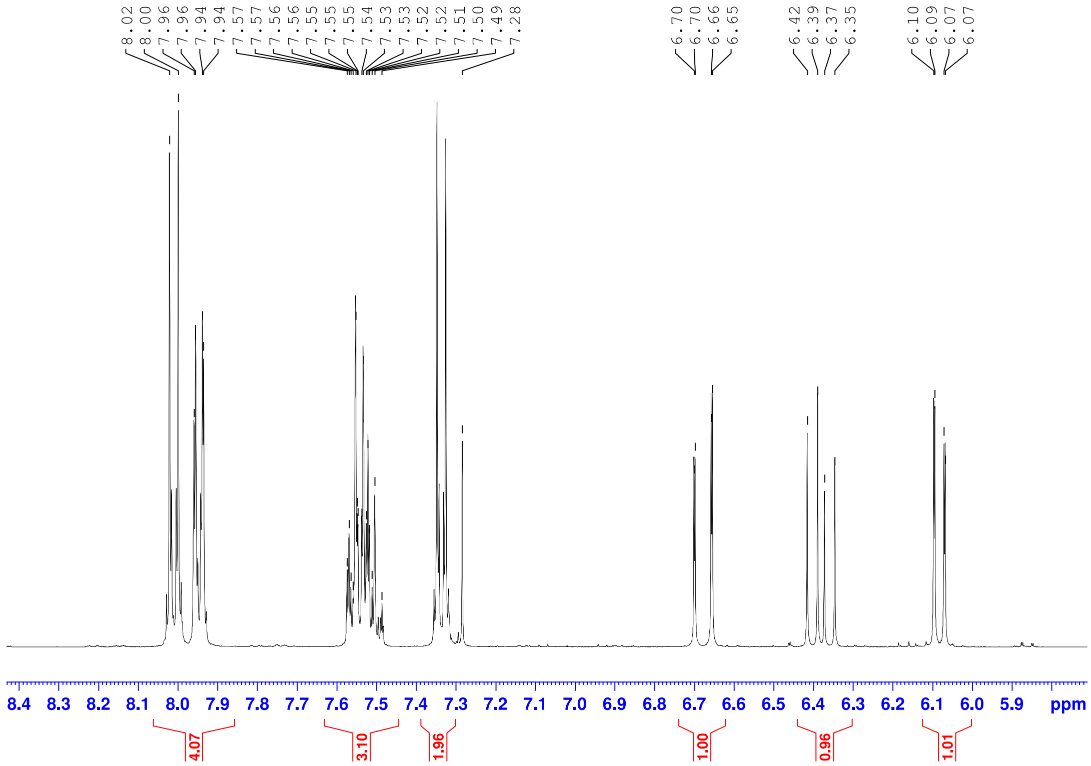

Figure S8. 400 MHz ($CDCl_3$)^{1}H NMR spectrum of monomer **M1b**.

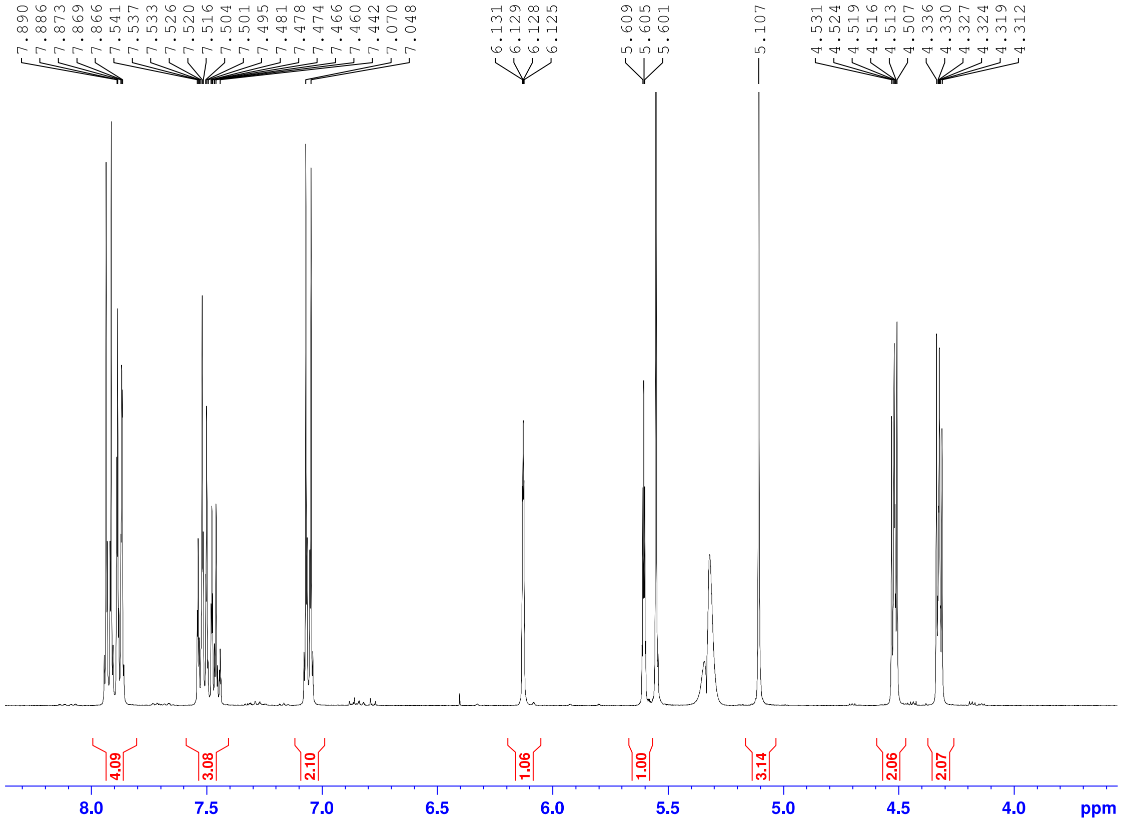

Figure S9. 400 MHz ($CDCl_3$)^{1}H NMR spectrum of monomer **M2a**.

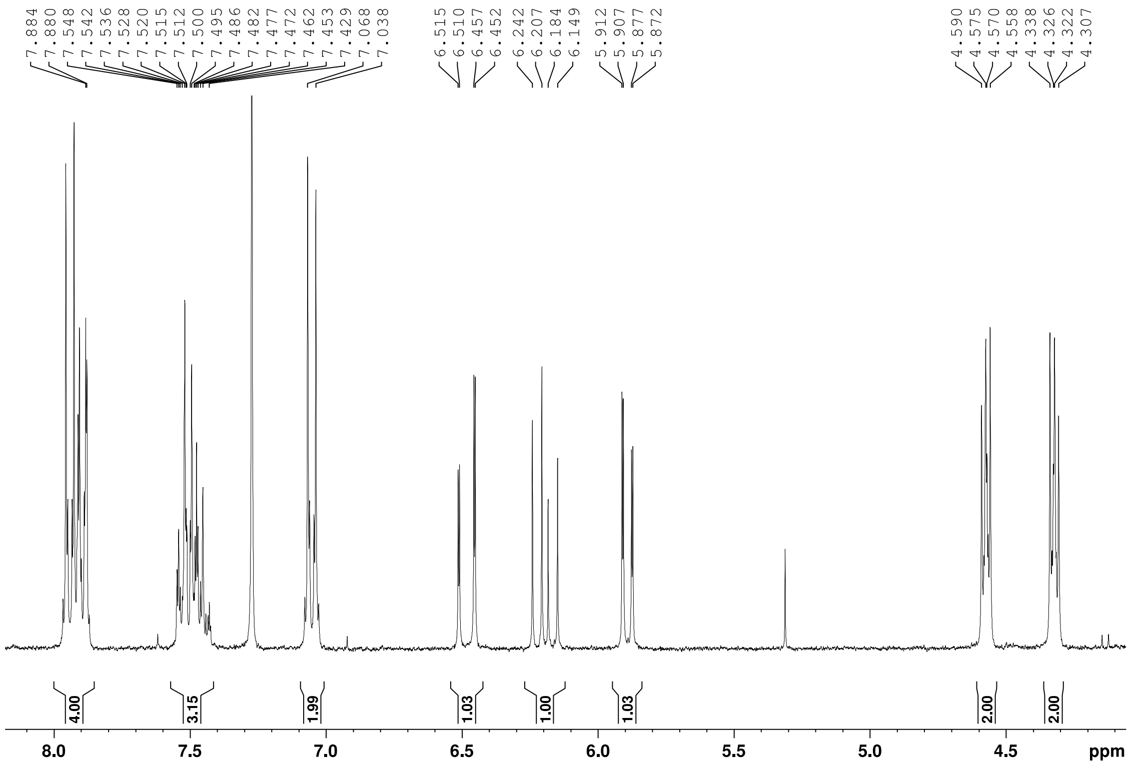

Figure S10. 400 MHz ($CDCl_3$)^{1}H NMR spectrum of monomer **M2b**.

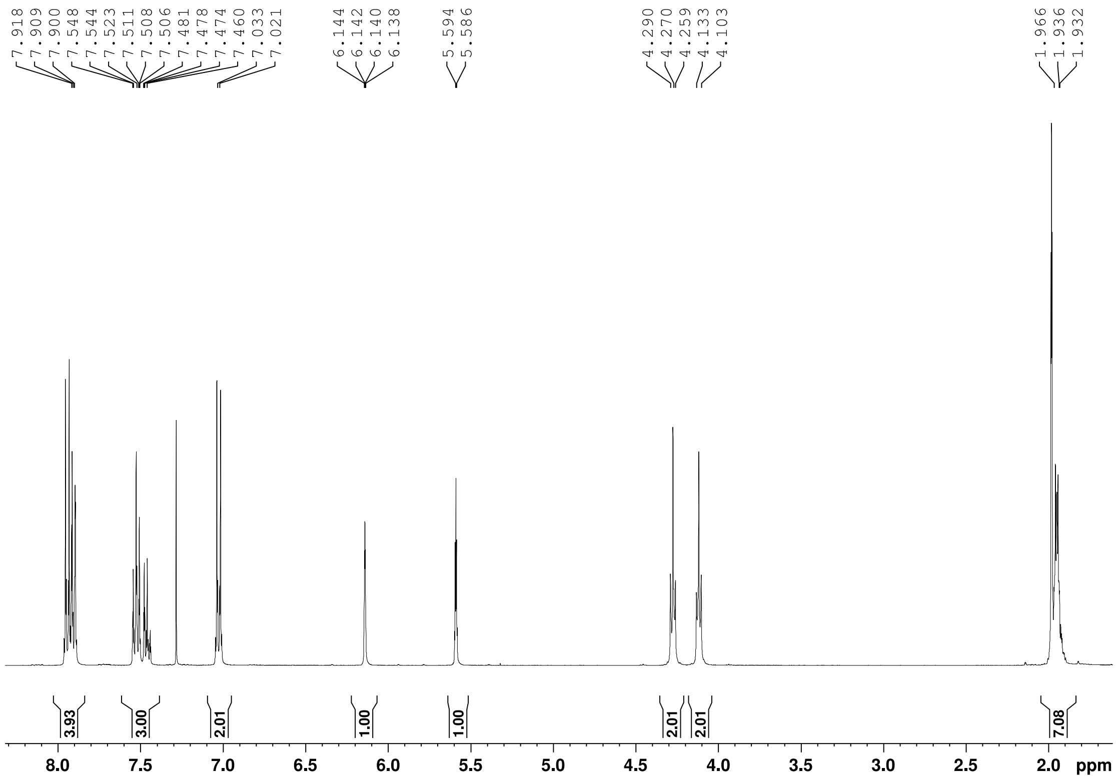

Figure S11. 400 MHz ($CDCl_3$)^{1}H NMR spectrum of monomer **M3a**.

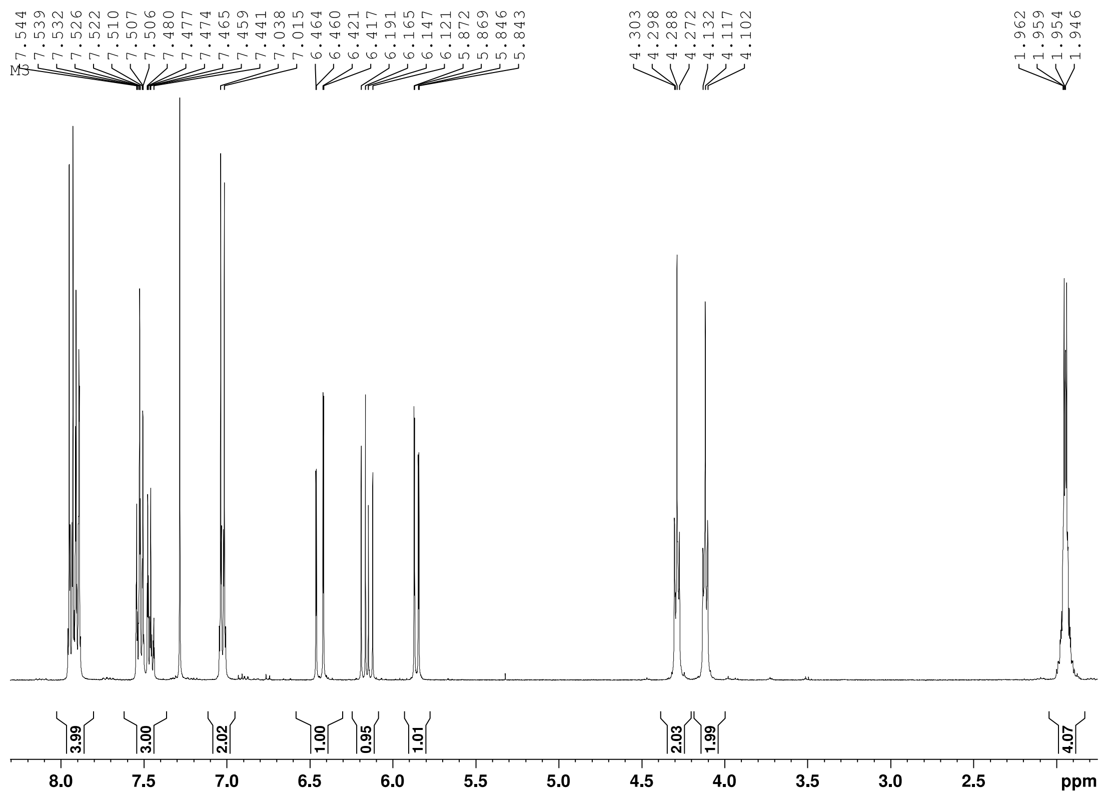

Figure S12. 400 MHz ($CDCl_3$)^{1}H NMR spectrum of monomer **M3b**.

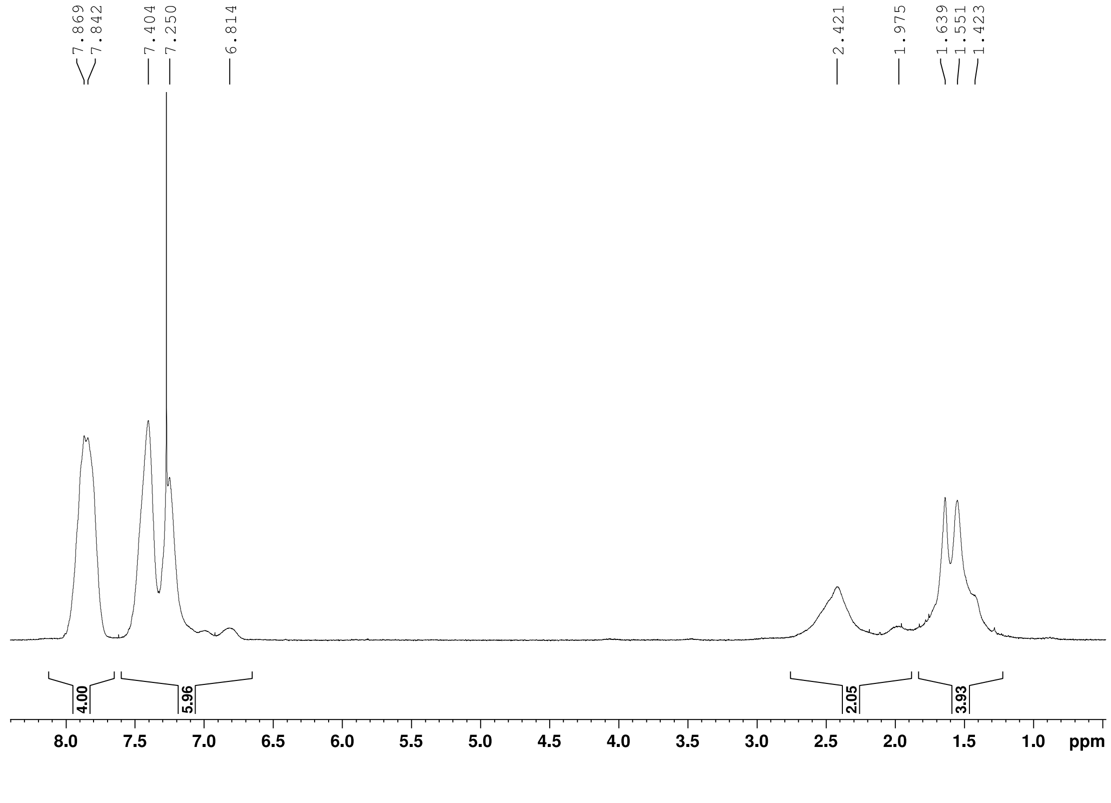

Figure S13. 400 MHz ($CDCl_3$)^{1}H NMR spectrum of polymer **1a**.

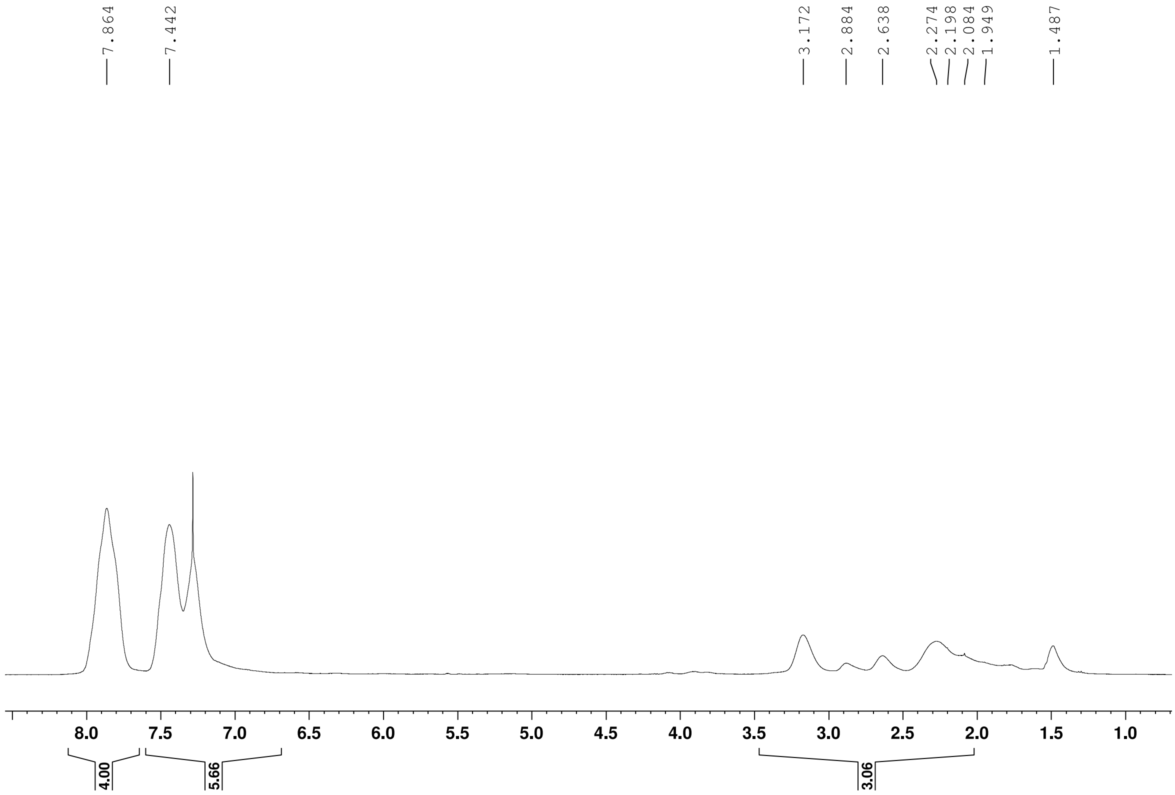

Figure S14. 400 MHz ($CDCl_3$)^{1}H NMR spectrum of polymer **1b**.

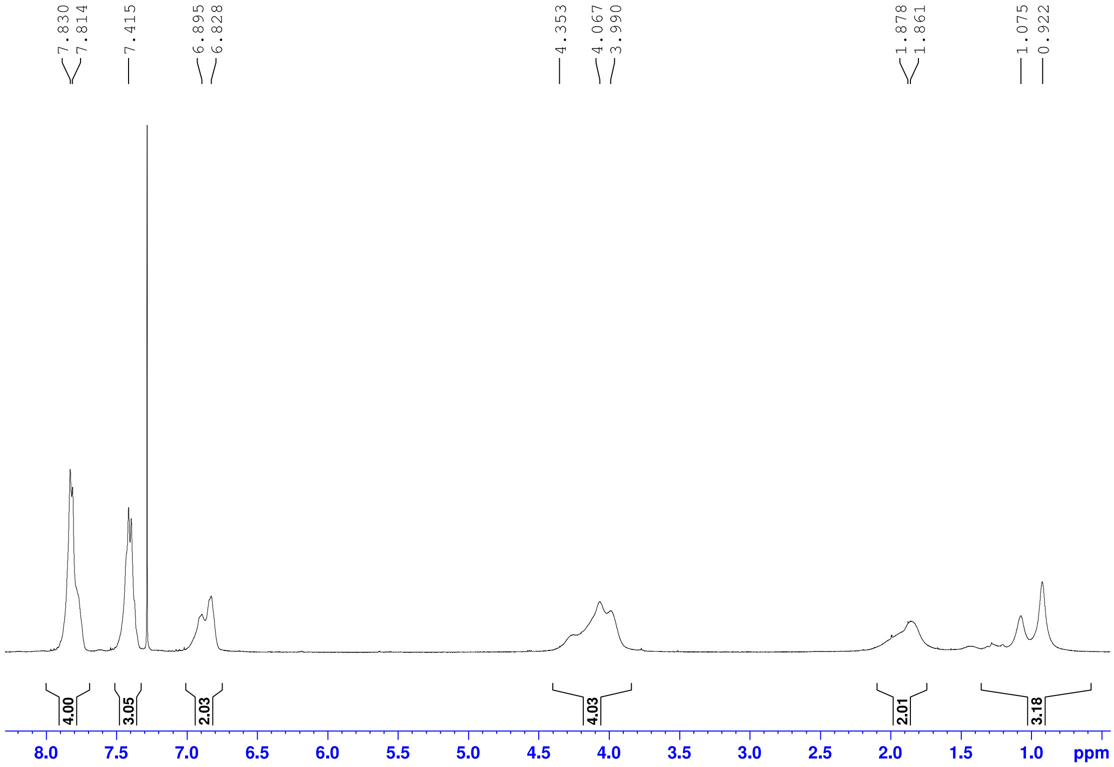

Figure S15. 400 MHz ($CDCl_3$)^{1}H NMR spectrum of polymer **2a**.

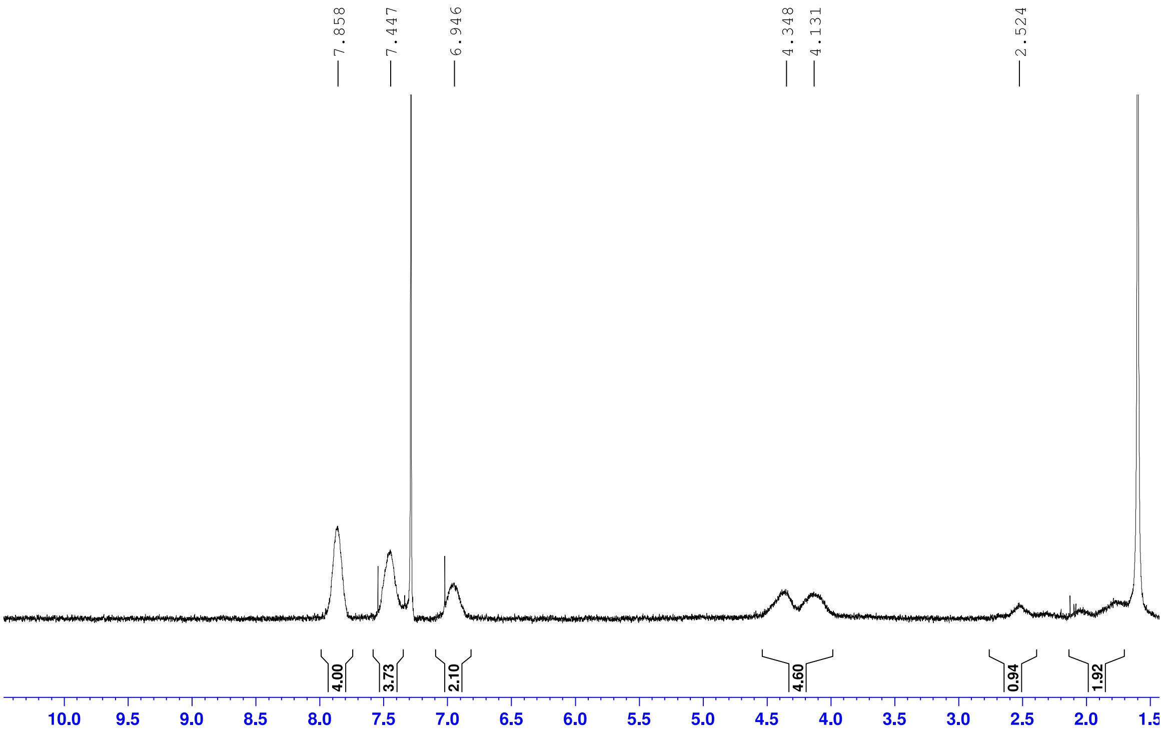

Figure S16. 400 MHz ($CDCl_3$)^{1}H NMR spectrum of polymer **2b**.

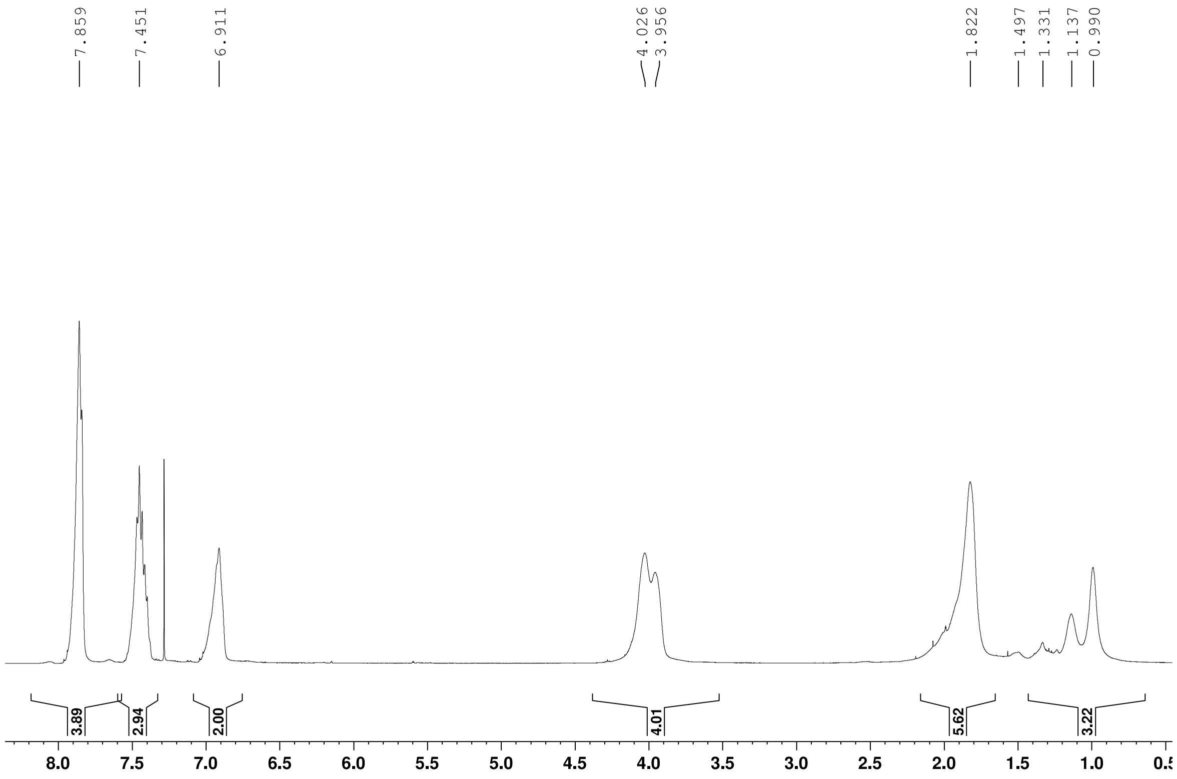

Figure S17. 400 MHz ($CDCl_3$)^{1}H NMR spectrum of polymer **3a**.

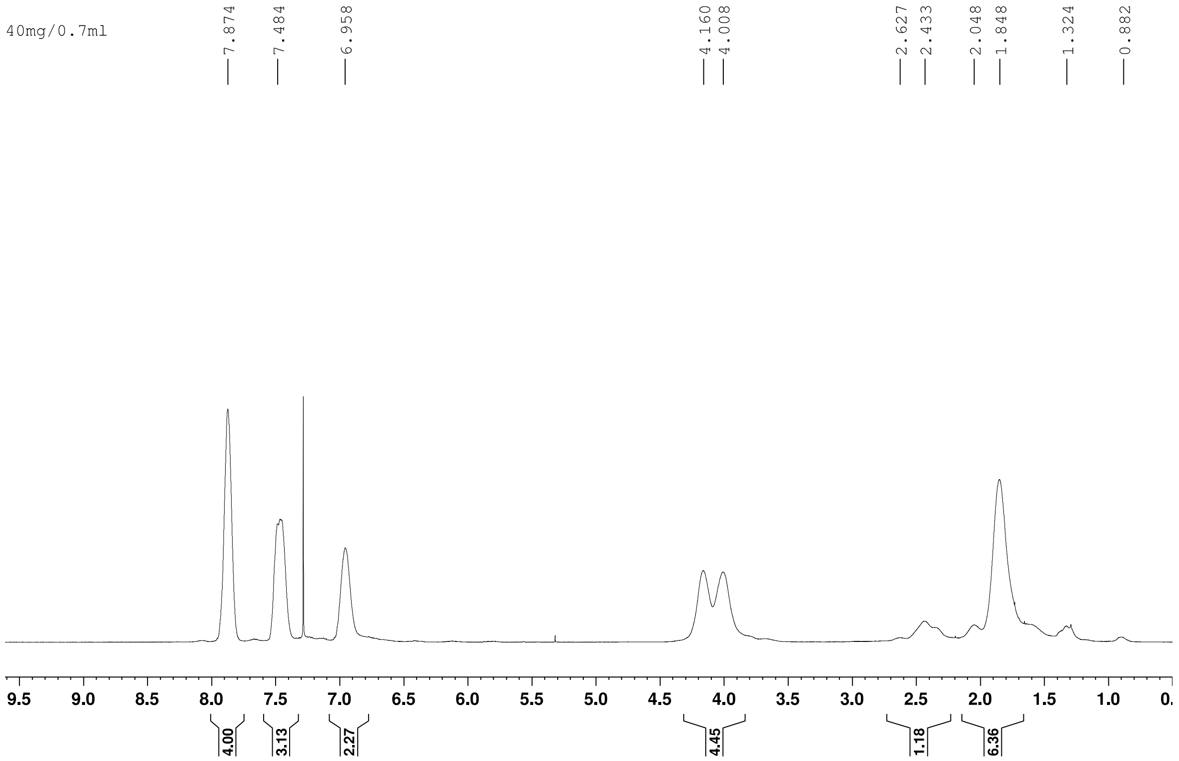

Figure S18. 400 MHz ($CDCl_3$)^{1}H NMR spectrum of polymer **3b**.

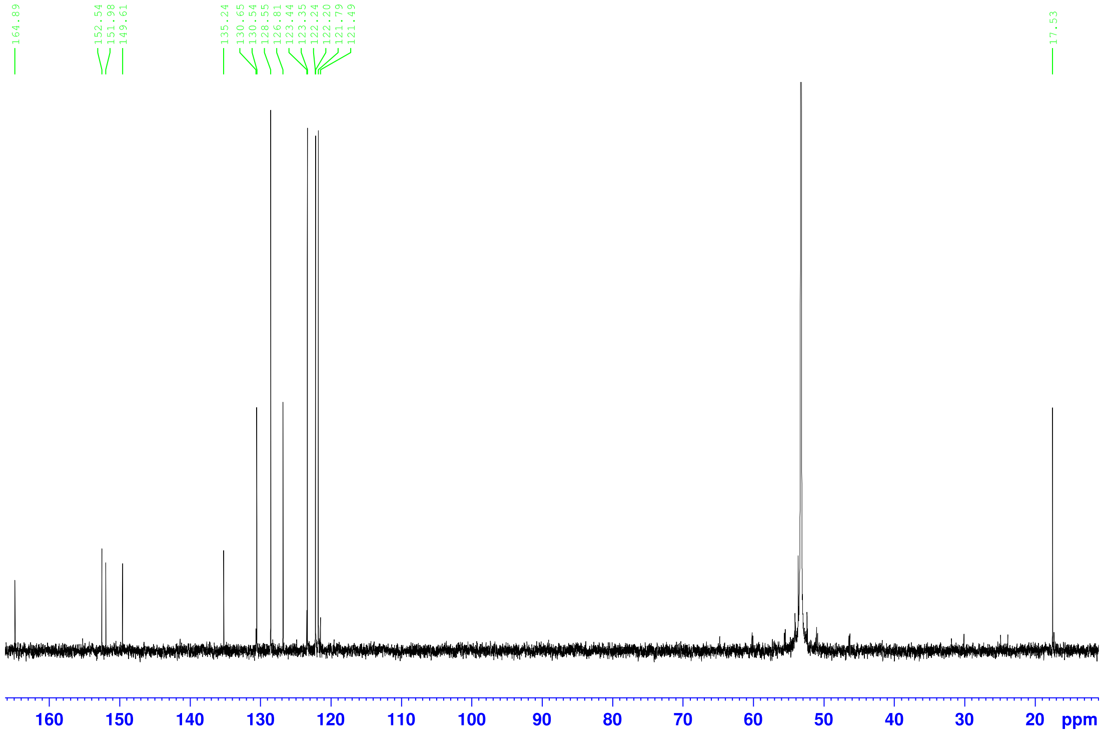

Figure S19. 100 MHz ($CDCl_3$)^{13}C NMR spectrum of monomer **1a**.

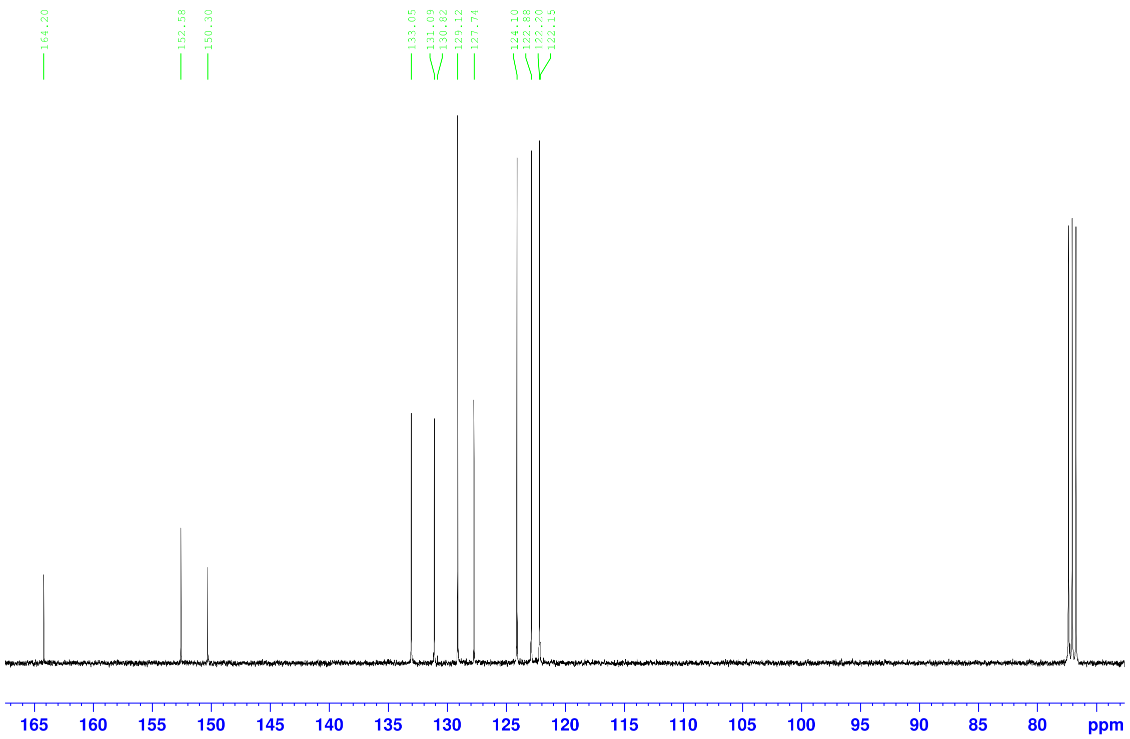

Figure S20. 100 MHz ($CDCl_3$)^{13}C NMR spectrum of monomer **1b**.

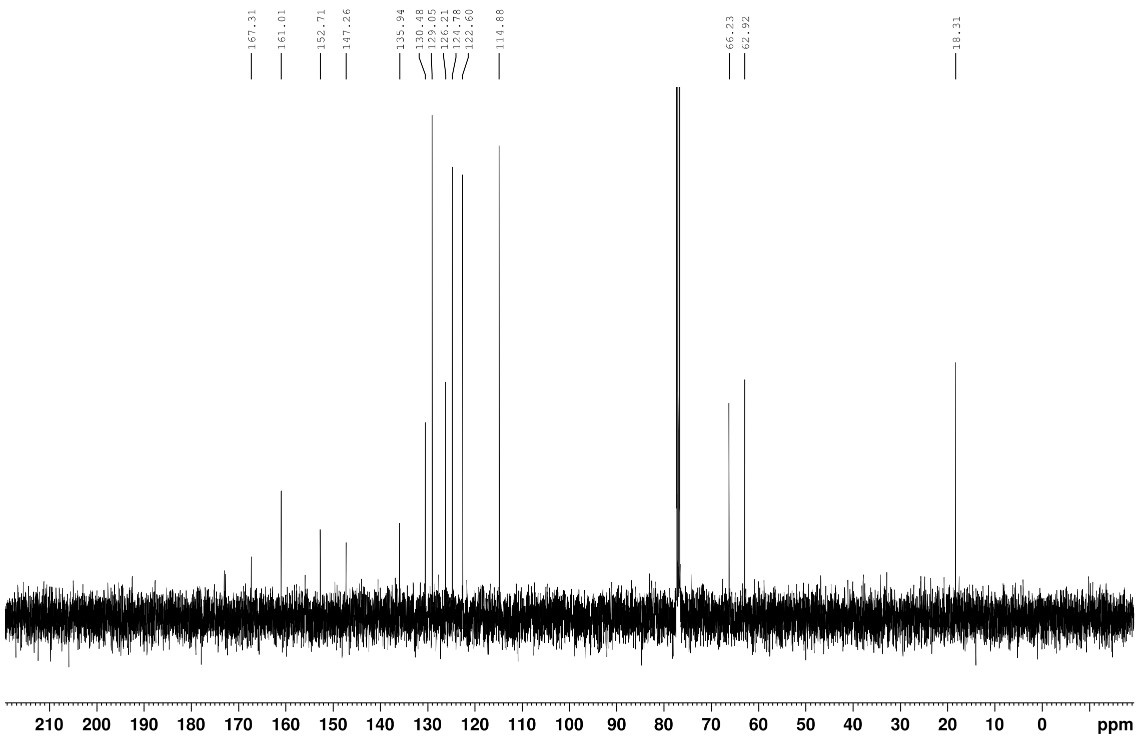

Figure S21. 100 MHz ($CDCl_3$) ^{13}C NMR spectrum of monomer **2a**.

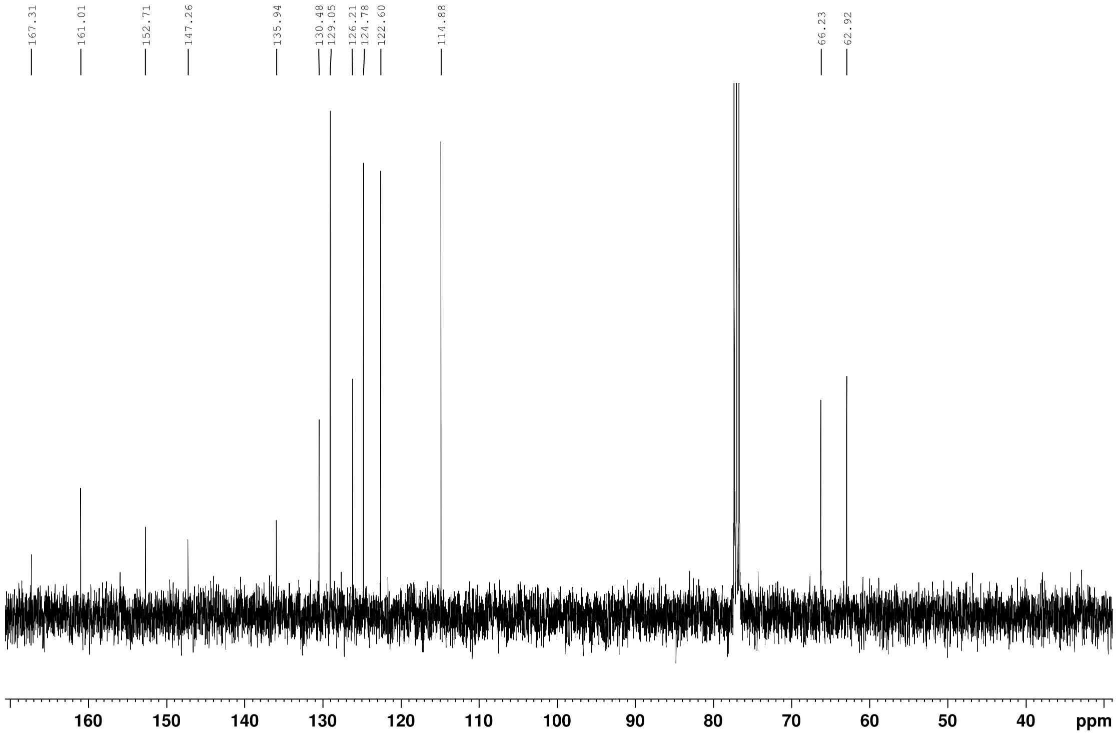

Figure S22. 100 MHz ($CDCl_3$) ^{13}C NMR spectrum of monomer **2b**.

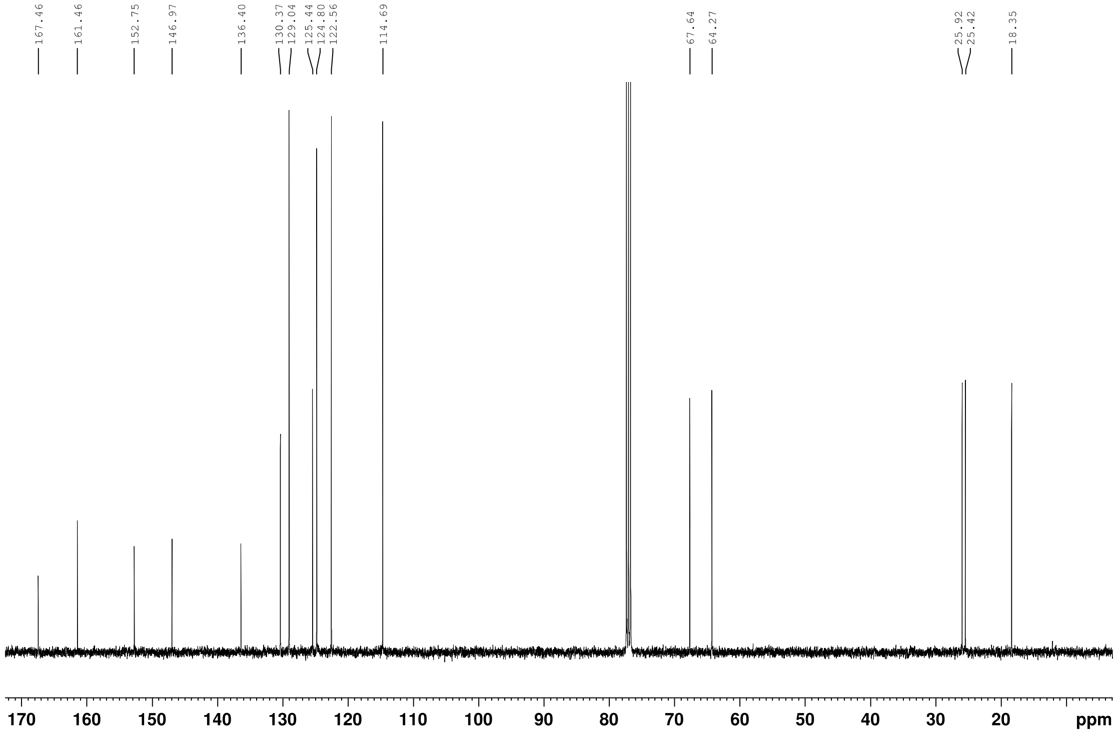

Figure S23. 100 MHz ($CDCl_3$) ^{13}C NMR spectrum of monomer **3a**.

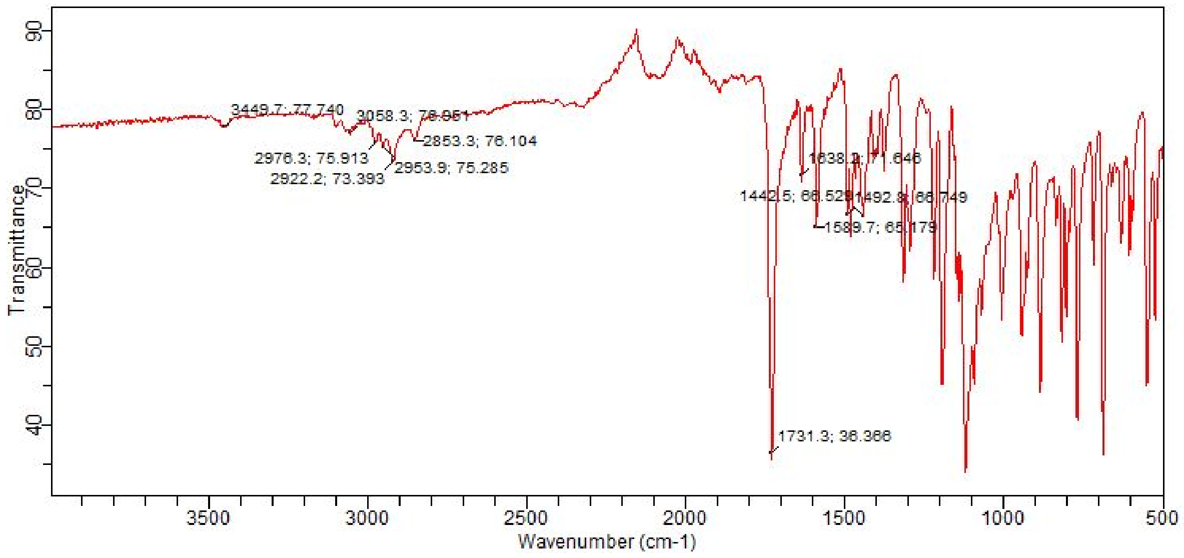

Figure S24. IR spectrum of monomer **1a**.

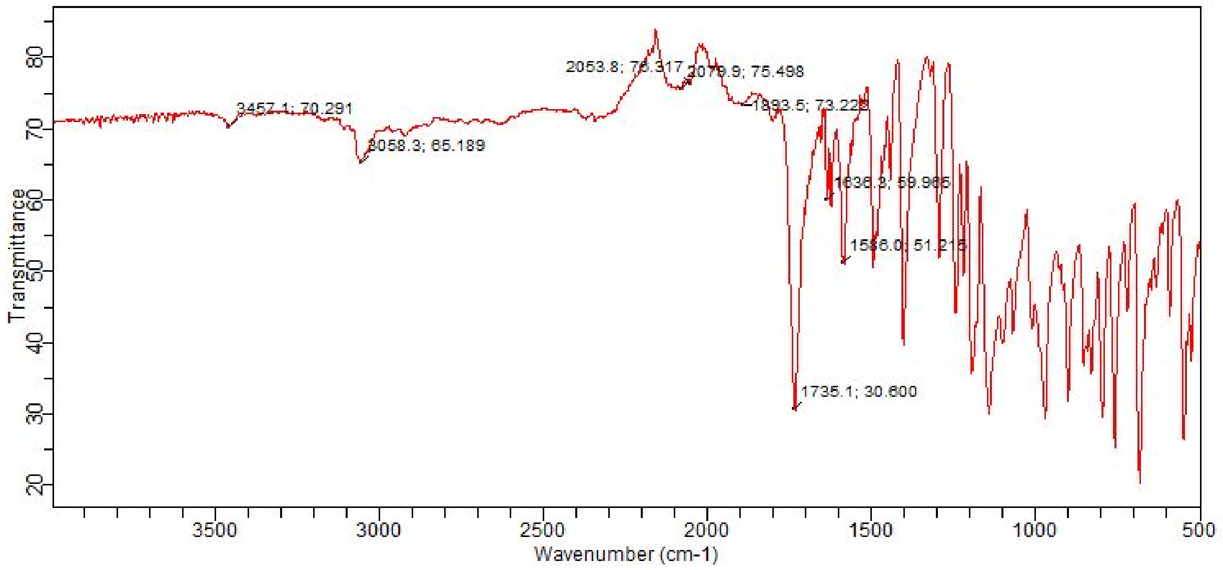

Figure S25. IR spectrum of monomer **1b**.

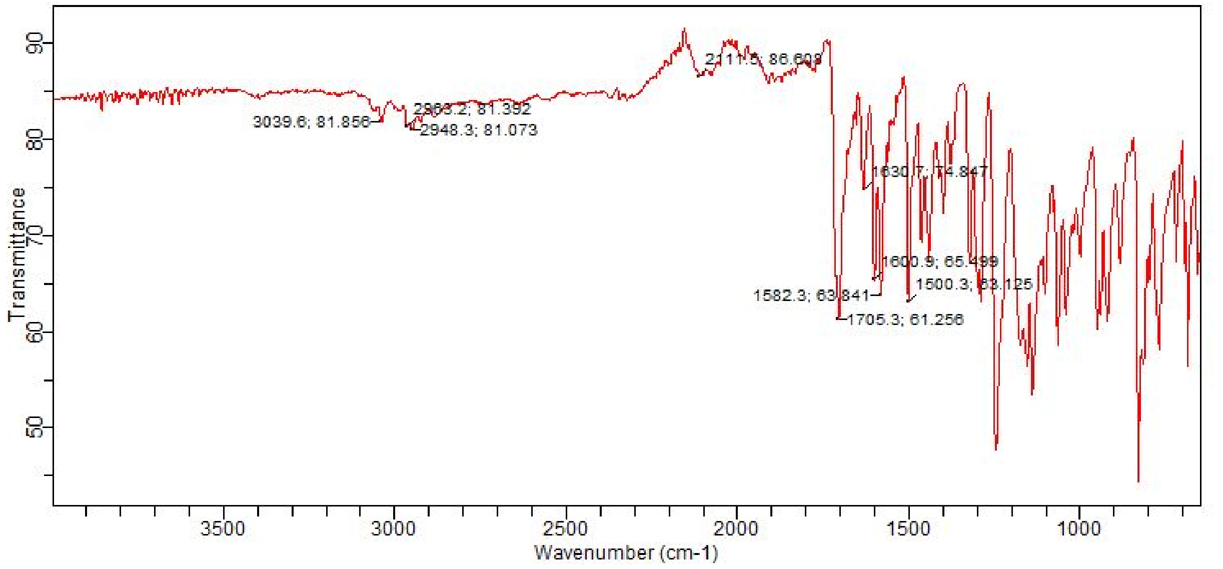

Figure S26. IR spectrum of monomer **2a**.

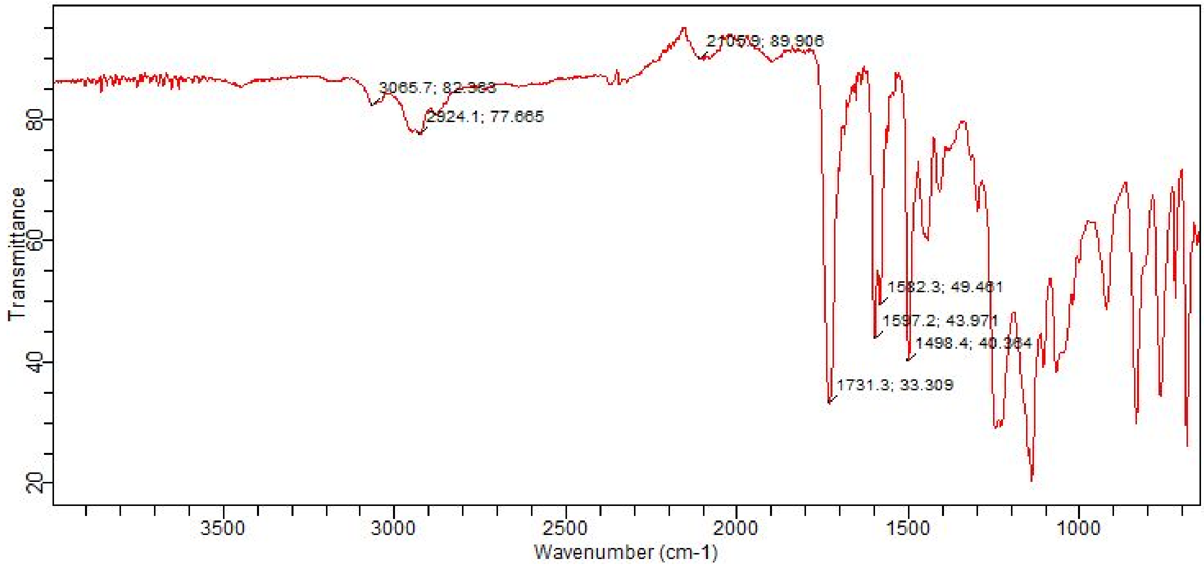

Figure S27. IR spectrum of monomer **2b**.

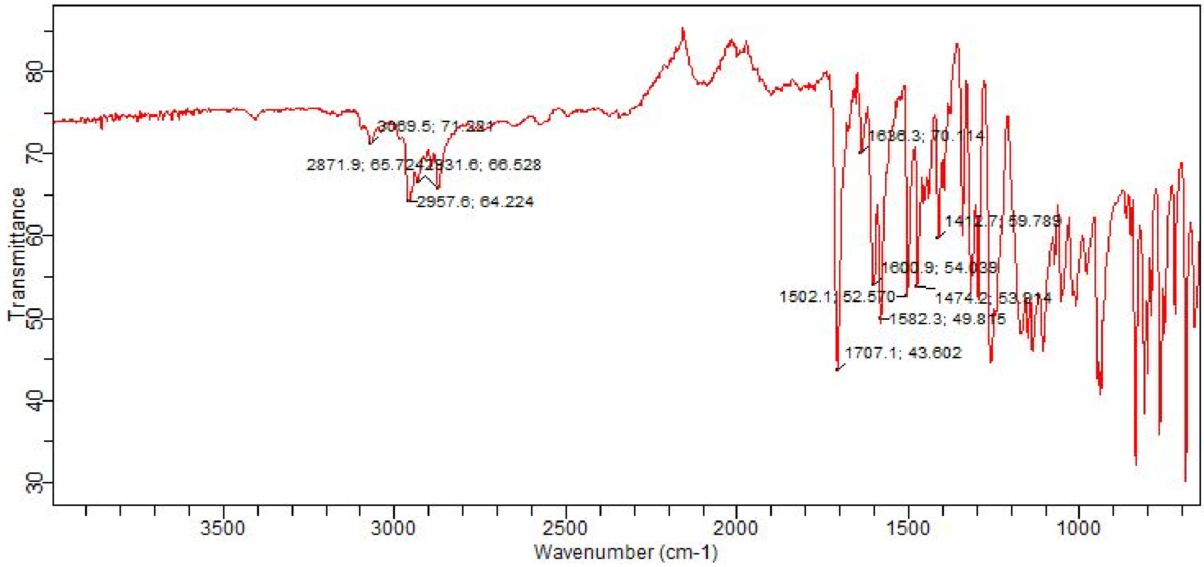

Figure S28. IR spectrum of monomer **3a**.

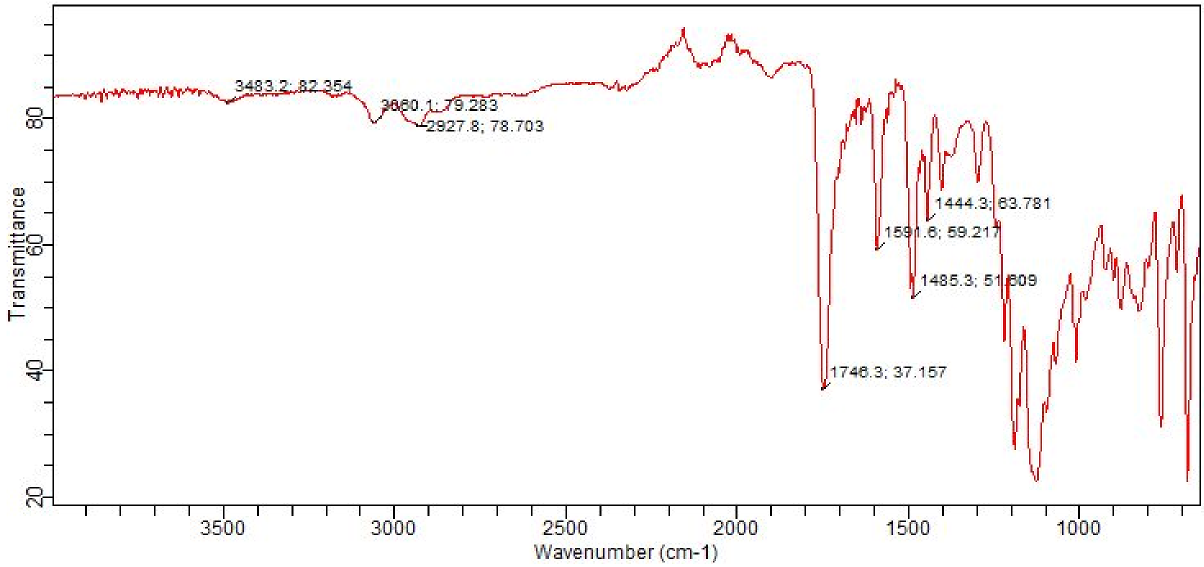

Figure S29. IR spectrum of monomer **3b**.

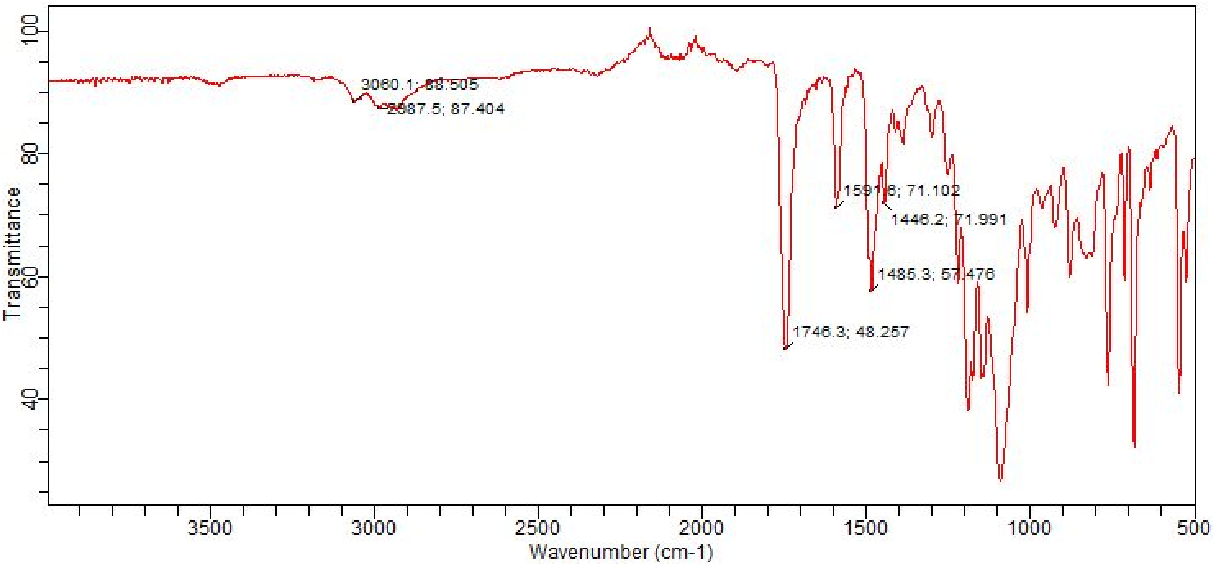

Figure S30. IR spectrum of polymer **1a**.

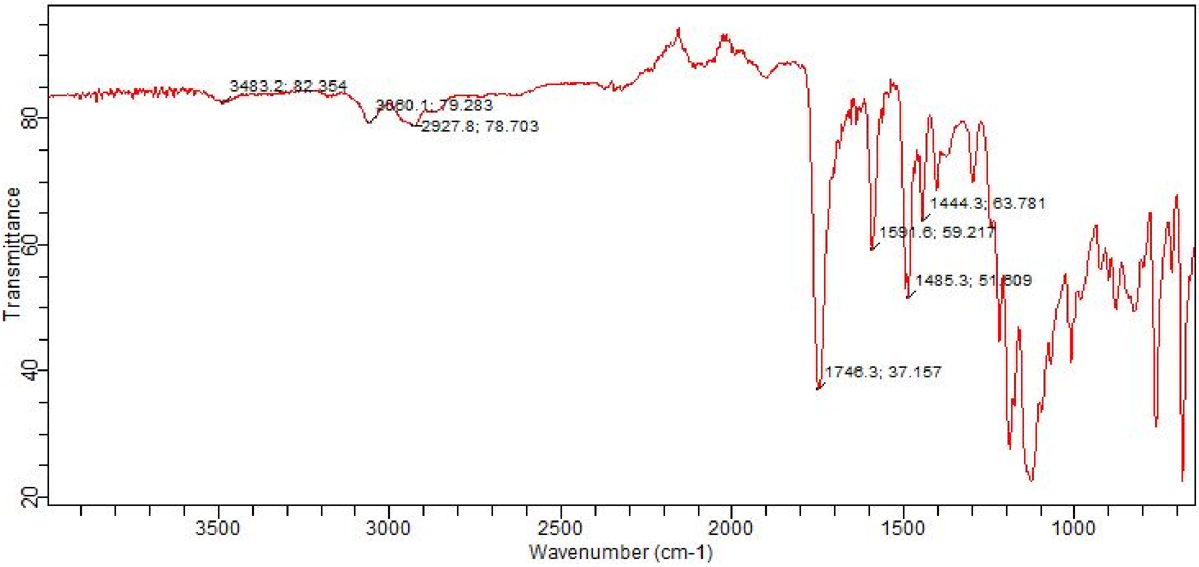

Figure S31. IR spectrum of polymer **1b**.

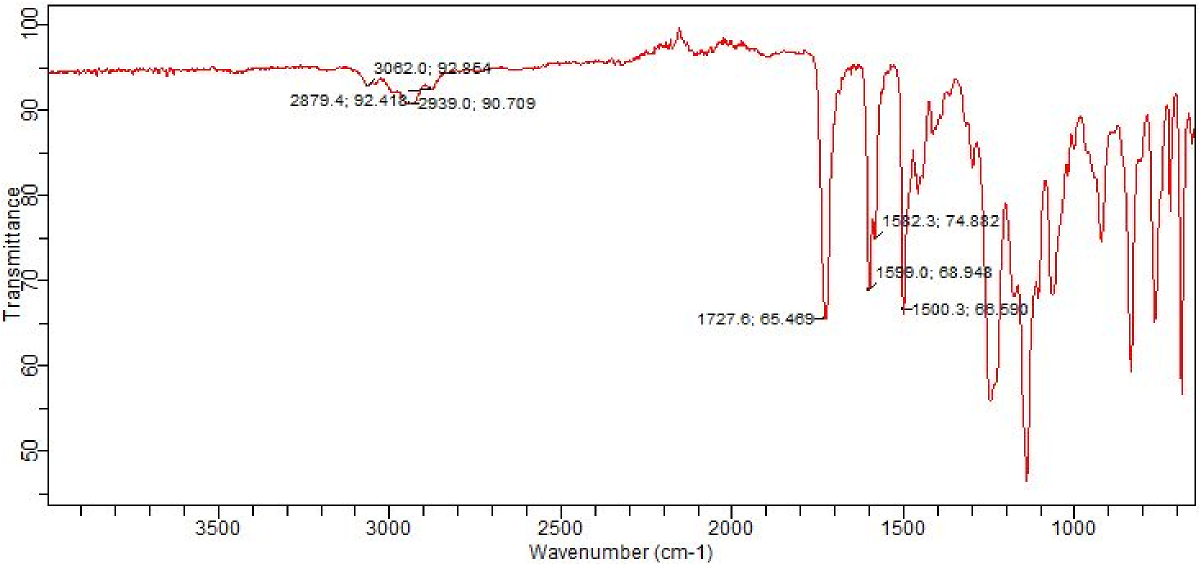

Figure S32. IR spectrum of polymer **2a**.

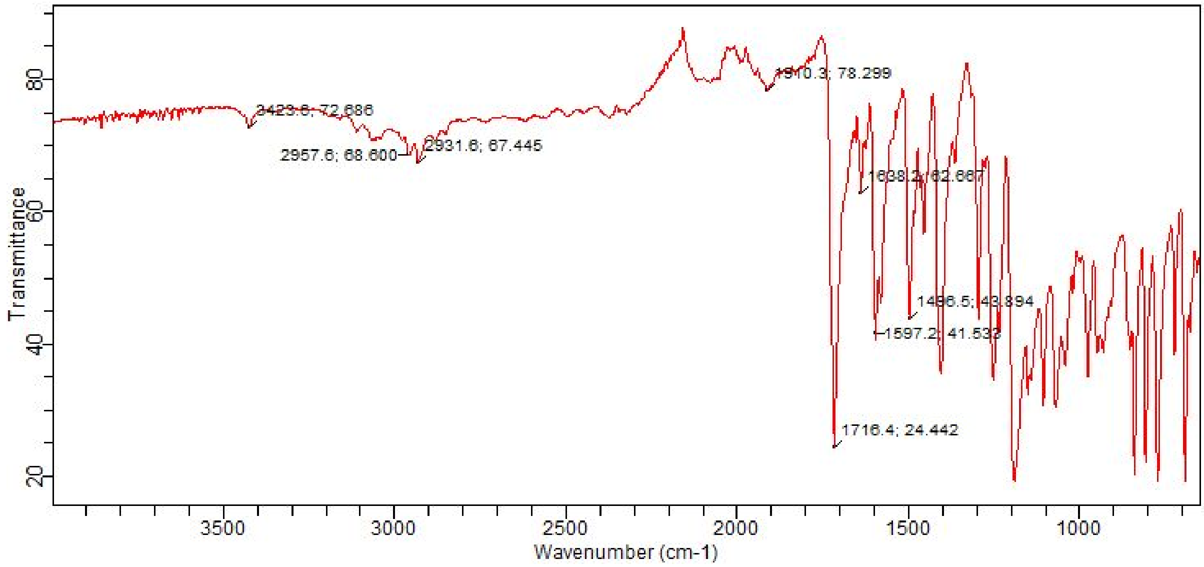

Figure S33. IR spectrum of polymer **2b**.

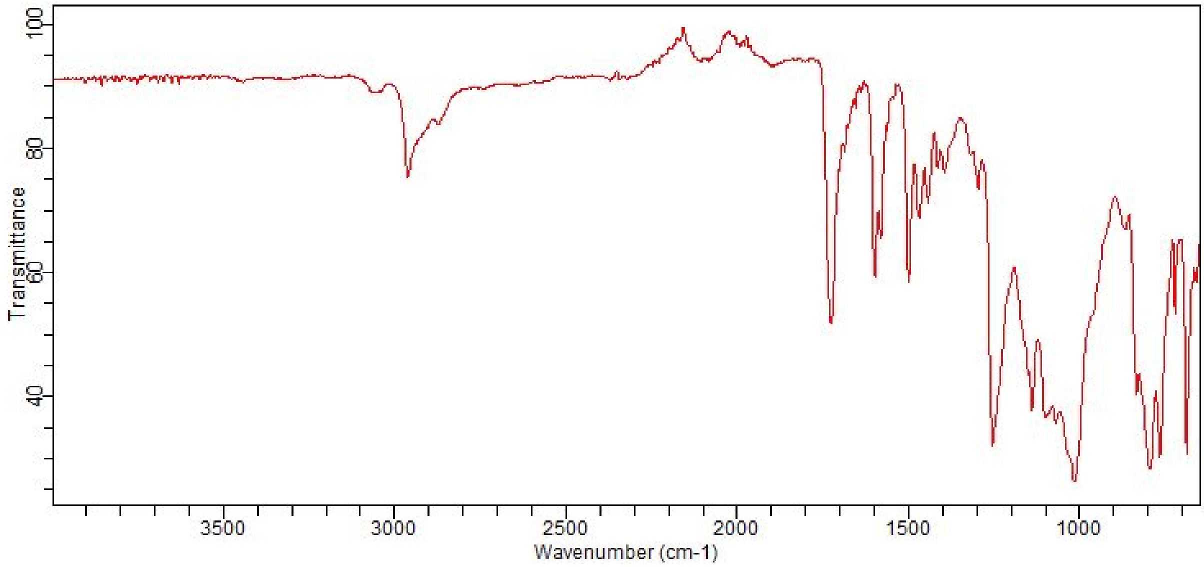

Figure S34. IR spectrum of polymer **3a**.

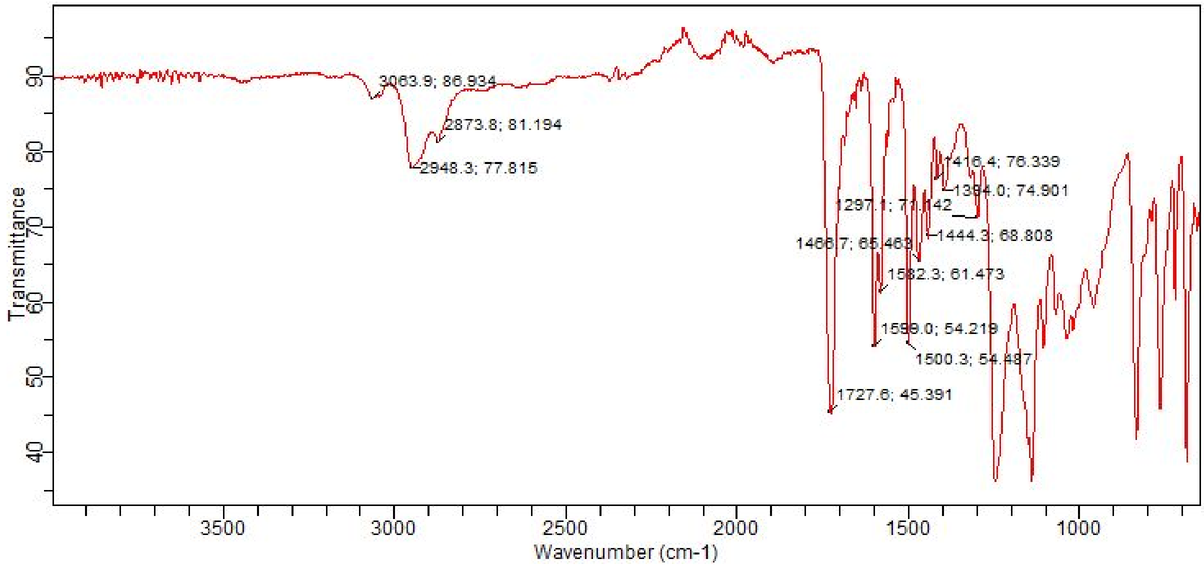

Figure S35. IR spectrum of polymer **3b**.

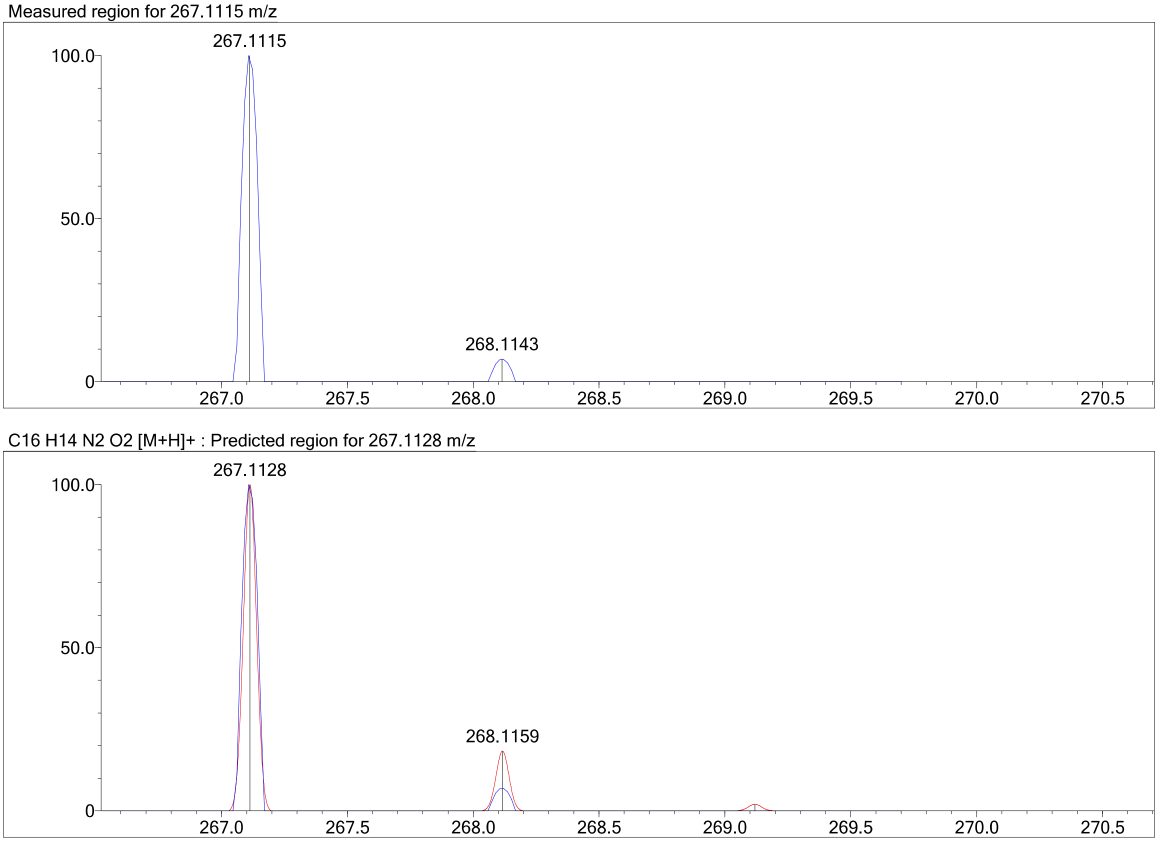

Rank	Score	Formula (M)	Ion	Meas. m/z	Pred. m/z	Df. (mDa)	Df. (ppm)	Iso	DBE
16	58.08	C16 H14 N2 O2	[M+H]+	267.1115	267.1128	-1.3	-4.87	64.30	11.0

Figure S36. ESI spectrum of monomer **1a**.

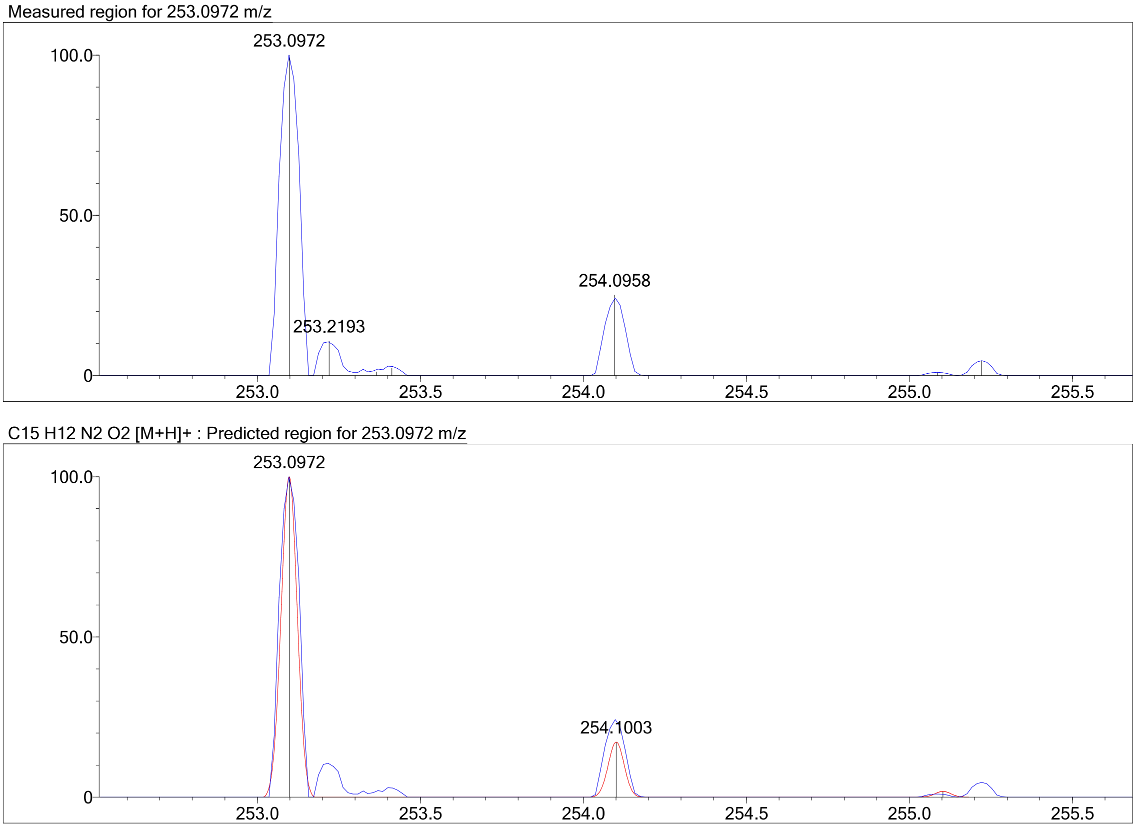

Rank	Score	Formula (M)	Ion	Meas. m/z	Pred. m/z	Df. (mDa)	Df. (ppm)	Iso	DBE
1	61.00	C15 H12 N2 O2	[M+H]+	253.0972	253.0972	0.0	0.00	61.00	11.0

Figure S37. APCI spectrum of monomer **1b**.

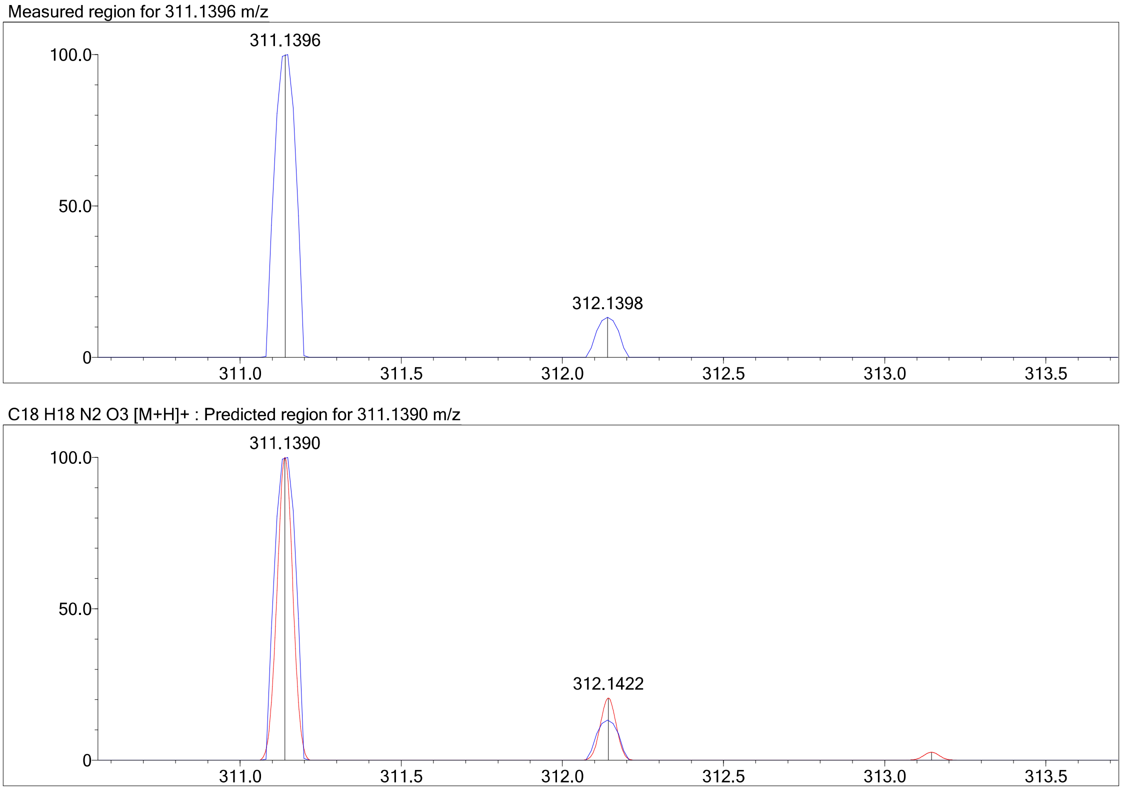

Rank	Score	Formula (M)	Ion	Meas. m/z	Pred. m/z	Df. (mDa)	Df. (ppm)	Iso	DBE
1	71.99	C18 H18 N2 O3	[M+H]+	311.1396	311.1390	0.6	1.93	73.70	11.0

Figure S38. APCI spectrum of monomer **2a**.

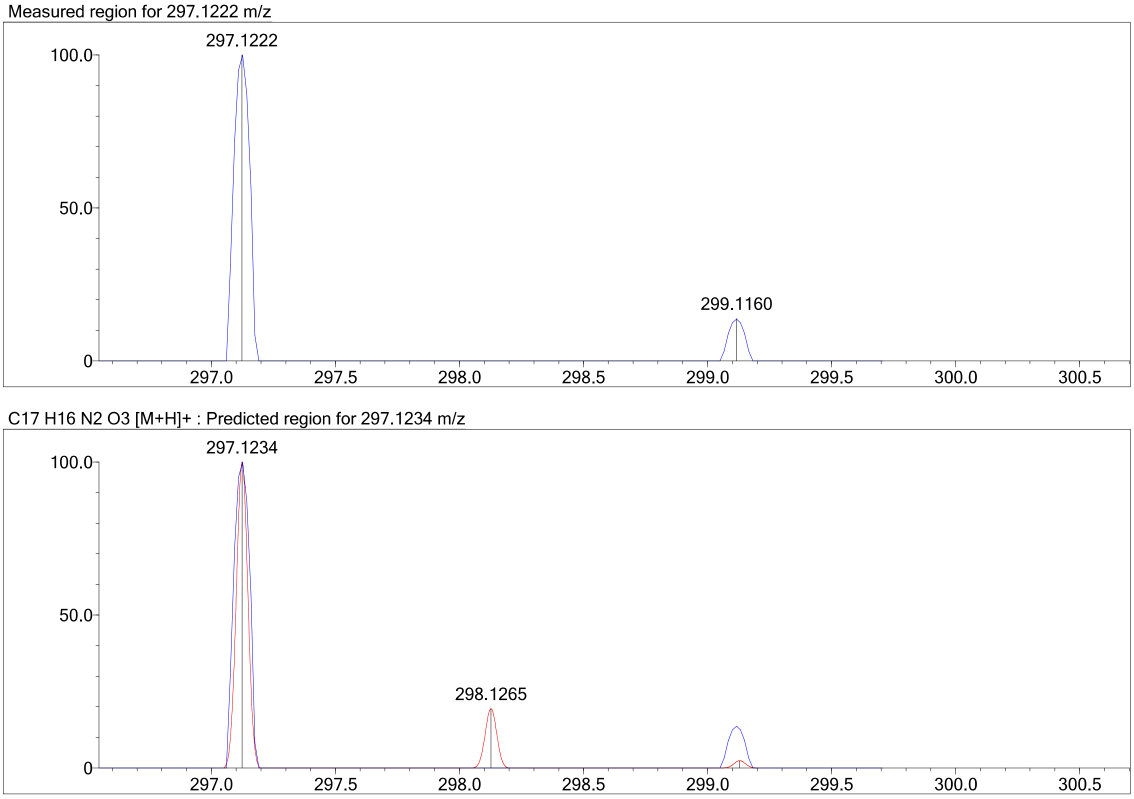

Rank	Score	Formula (M)	Ion	Meas. m/z	Pred. m/z	Df. (mDa)	Df. (ppm)	Iso	DBE
4	35.81	C17 H16 N2 O3	[M+H]+	297.1222	297.1234	-1.2	-4.04	38.76	11.0

Figure S39. APCI spectrum of monomer **2b**.

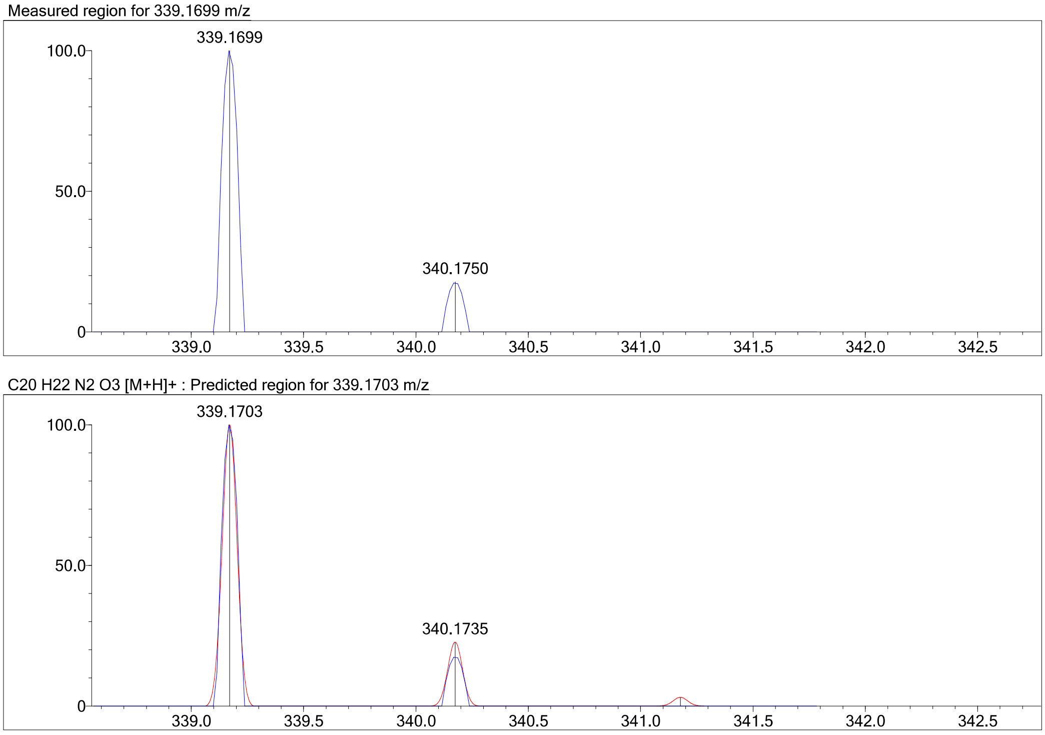

Rank	Score	Formula (M)	Ion	Meas. m/z	Pred. m/z	Df. (mDa)	Df. (ppm)	Iso	DBE
4	79.71	C20 H22 N2 O3	[M+H]+	339.1699	339.1703	-0.4	-1.18	80.07	11.0

Figure S40. APCI spectrum of monomer **3a**.

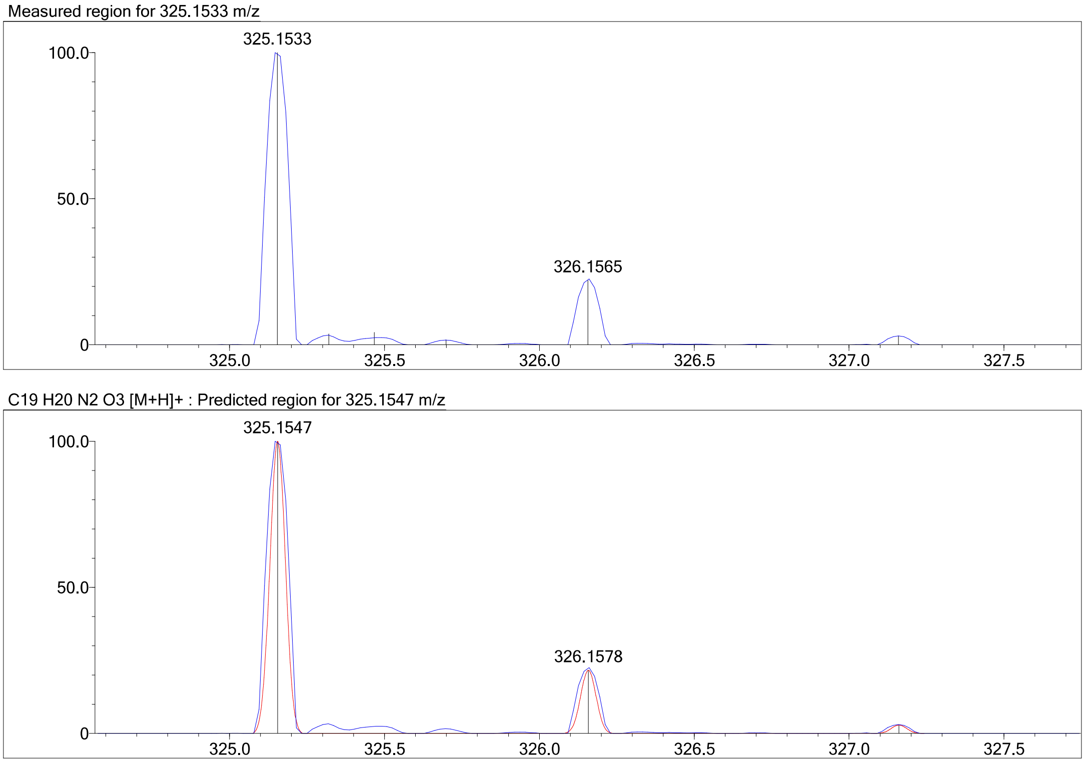

Rank	Score	Formula (M)	Ion	Meas. m/z	Pred. m/z	Df. (mDa)	Df. (ppm)	Iso	DBE
5	91.72	C19 H20 N2 O3	[M+H]+	325.1533	325.1547	-1.4	-4.31	100.00	11.0

Figure S41. APCI spectrum of monomer **3b**.

9. References

(1) Moniruzzaman, M.; Sabey, C. J.; Fernando, G. F. Synthesis of Azobenzene-Based Polymers and the in-Situ Characterization of Their Photoviscosity Effects. *Macromolecules* **2004**, *37* (7).
(2) Su, X.; Xiao, C.; Hu, C. Facile Preparation and Dual Responsive Behaviors of Starch-Based Hydrogel Containing Azo and Carboxylic Groups. *Int. J. Biol. Macromol.* **2018**, *115*, 1189–1193.
(3) Kehe, G. M.; Mori, D. I.; Schurr, M. J.; Nair, D. P. Optically Responsive, Smart Anti-Bacterial Coatings via the Photofluidization of Azobenzenes. *ACS Appl. Mater. Interfaces* **2019**, *11* (2), 1760–1765.
(4) Ding, L.; Li, J.; Wang, C.; Lin, L. Controlled Synthesis of Photosensitive Graft Copolymers with High Azobenzene-Chromophore Loading Densities in the Main and Side Chains by Combining ATRP and ADMET Polymerization. *React. Funct. Polym.* **2015**, *91–92*, 85–92.
(5) Sabey, C. J.; Moniruzzaman, M.; Fernando, G. F.; Badcock, R. A.; Winter, D.; Akhavan, J.; Kronfli, E.; Bandara, A. In-Situ Characterisation of Photo-Actuating and Photo-Rheological Polymers. *MRS Online Proc. Libr.* **2004**, *785* (1), 88.
(6) Barmatov, E. B.; Medvedev, A. V.; Ivanov, S. A. Influence of the Molecular Structure of Comb-Shaped Photochromic Copolymers on Photoinduced Birefringence. *Russ. J. Appl. Chem.* **2004**, *77* (4), 608–612.
(7) Zhitomirsky, D.; Cho, E.; Grossman, J. C. Solid-State Solar Thermal Fuels for Heat Release Applications. *Adv. Energy Mater.* **2016**, *6* (6), 1502006.
(8) Abrakhi, S.; Péralta, S.; Fichet, O.; Teyssié, D.; Cantin, S. Poly(Azobenzene Acrylate-Co-Fluorinated Acrylate) Spin-Coated Films: Influence of the Composition on the Photo-Controlled Wettability. *Langmuir* **2013**, *29* (30), 9499–9509.
